# Can Generic Medications Be a Safe and Effective Alternative to Brand-Name Drugs for Cardiovascular Disease Treatment? A Systematic Review and Meta-Analysis

**DOI:** 10.31083/RCM26116

**Published:** 2025-03-07

**Authors:** Bing Luo, Feng Yu, Weihong Ge, Xian Yang

**Affiliations:** ^1^Department of Pharmacy, Nanjing Drum Tower Hospital, Affiliated Hospital of Medical School, Nanjing University, 210008 Nanjing, Jiangsu, China; ^2^School of Basic Medicine and Clinical Pharmacy, China Pharmaceutical University, 210009 Nanjing, Jiangsu, China

**Keywords:** generic drug, brand-name drug, cardiovascular diseases, meta-analysis, efficacy, safety

## Abstract

**Background:**

Cardiovascular disease is the leading cause of death in most of the world. Previous meta-analyses of generic drugs for the treatment of cardiovascular disease have not provided sufficient evidence to demonstrate the true efficacy and safety of the drugs. Subsequently, concern exists regarding whether the use of generic drugs can fully substitute brand-name drugs in clinical treatment. To enhance the evidence for generic drugs, this meta-analysis compares the actual effectiveness of generic drugs with brand-name drugs in preventing and treating cardiovascular diseases. This study aimed to resolve the controversy over whether generic drugs in cardiovascular disease can replace brand-name drugs, fully evaluating the best evidence on the clinical equivalence of generic drugs.

**Methods:**

The PubMed, Embase, The Cochrane Library, and Clinicaltrials.gov databases were searched. The search period included articles published before December 2023. Studies on generic and branded cardiovascular drugs were collected, and two independent reviewers screened eligibility, extracted study data, and assessed the risk of bias. Safety outcomes included major adverse cardiovascular events and other adverse events. Efficacy outcomes included relevant vital signs (e.g., blood pressure, heart rate, urine volume) and laboratory measures (e.g., international normalized ratio, low-density lipoprotein cholesterol, platelet aggregation inhibition). A meta-analysis and subgroup analysis were conducted using the Rev Man software.

**Results:**

A total of 4238 studies were retrieved, and 87 studies (n = 2,303,818) were included in the qualitative analysis. There were 57 quantitatively assessed studies (n = 560,553), including angiotensin II receptor blockers, beta-blockers, calcium channel blockers, antithrombotic drugs (anticoagulants or antiplatelet agents), diuretics, statins, and other classes of cardiovascular medications. Regarding clinical safety, 19 studies assessed the occurrence of major adverse cardiovascular events (MACEs) (n = 384,640), and 35 reported secondary adverse events (n = 580,125). In addition to the MACEs for statins (risk ratio (RR) 1.13 [1.05, 1.21]) and adverse events (AEs) for calcium channel blockers (RR 0.90 [0.88, 0.91]), there were no significant differences in the overall risk of MACEs (RR = 1.02 [0.90, 1.15]) and minor adverse events (RR = 0.98 [0.91, 1.05]) between generic and brand-name cardiovascular drugs. In terms of effectiveness, there were no significant differences observed between the two groups in blood pressure (BP), platelet aggregation inhibition (PAI), international normalized ratio (INR), low-density lipoprotein (LDL), and urinary sodium levels. Subgroup analyses for the region, study design, duration of follow-up, and grant funding revealed no significant differences in the risk of MACEs. However, the risk of AE was significantly higher in the Asian region for brand-name cardiovascular drugs than for generics. There was no statistically significant difference in risk between generic and brand-name drugs in the remaining subgroup analyses.

**Conclusions:**

Cardiovascular drugs encompass many types; a minority of generic and brand-name drugs have discrepancies. Given the overall development trend of multi-manufacturer generic drugs in the future, this study provides a strong basis for the global application of generic drugs. The feasibility of generic drugs in terms of efficacy and safety in cardiovascular diseases is clarified. However, some drugs still need to be improved to replace the original drugs used in clinical practice completely. Therefore, large-sample, multicenter, high-quality studies are still required to guide the clinical use of cardiovascular drugs.

**The PROSPERO registration:**

CRD42023481597, https://www.crd.york.ac.uk/PROSPERO/view/CRD42023481597.

## 1. Introduction 

Cardiovascular disease (CVD) is a highly prevalent disease that affects morbidity and mortality worldwide. Global deaths related to cardiovascular disease have increased from 12.4 million in 1990 to 19.8 million in 2022, with actual CVD deaths rising significantly [1]. Between 2025 and 2050, there will be a further 90.0% increase in cardiovascular prevalence and a 73.4% increase in crude mortality, with an expected 35.6 million cardiovascular-related deaths in 2050 (from 20.5 million in 2025) [2]. CVD now accounts for approximately one-third of all deaths globally, and rational and effective pharmacological treatment is crucial for controlling disease progression. Currently, the global burden of CVD is classified as heavy. Generic drugs can alleviate the burden on patients, payers, and healthcare systems, offering a promising alternative to branded drugs [3]. Driven by policies in various regions worldwide, there has been a surge in the market share of generic drugs, followed by a gradual trend towards commercialization.

Traditional generic drugs are structurally and formulaically identical copies of brand-name drugs. Generic drugs are bioequivalent to the original brand, which is required for marketing approval of generic drugs. The mean values of the pharmacokinetic (PK) parameters are closely similar between generic and brand. The 95% confidence intervals (CIs) of the generic-to-drug ratio for key PK parameters (e.g., maximum concentration (C max) and area under the curve (AUC)) are required to lie within 80% and 125% of 1.00, which is the value that represents the ideal score [4]. However, bioequivalence does not imply that generic and brand-name drugs are interchangeable, and bioequivalence alone is insufficient to prove clinical equivalence. After switching to generic drugs, there were significant differences in clinical efficacy and safety compared to brand-name drugs [5], whereby users of generic drugs exhibited a relatively higher rate of hospital visits and an increase in reported adverse events [6]. A meta-analysis comparing the real-life clinical impact of brand-name and generic cardiovascular medications focused on all-cause hospital visits; however, the evidence provided was too diverse to draw definitive conclusions [7]. A further early meta-analysis included only randomized controlled trials (RCTs), with a larger proportion of studies in healthy individuals [8]. Moreover, a meta-analysis of branded and generic warfarin included 11 studies [9], while another meta-analysis compared branded versus generic clopidogrel in patients with cardiovascular disease and included only three prospective studies [10]. However, the number of studies included in the above analyses is deemed extremely limited and unconvincing. Most of the studies included previously were bioequivalence studies, which considered factors such as shorter study periods, smaller sample sizes, and physiological differences between healthy subjects and patients, meaning it is also challenging to demonstrate successfully the true effectiveness and safety of generic drugs. Therefore, it is impossible to answer whether generic medications are effective substitutes for brand-name drugs for therapeutic use.

The issue of the efficacy and safety of generic drugs is far-reaching, whereby previous instances of generic recalls and import bans have undermined confidence in using medicines [11]. Doctors, pharmacists, and patients continue to debate using generic drugs as alternatives; however, concerns regarding the quality and reliability of generic drugs persist, along with personal biases in favor of their use in reality [12, 13, 14]. A study based on real-world patient data have raised questions about the effectiveness of generics as substitutes for brand-name drugs [15]. Strong meta-analyses of relevant evidence for the large population of CVD patients remain limited, and systematic reviews based on existing evidence are especially necessary. Generic drugs have been in use for decades, and the findings and safety reports of studies on the use of cardiovascular medications are continually being updated. This review aims to synthesize the latest findings and data and perform a meta-analysis of the safety and effectiveness of generic drugs compared to brand-name drugs in treating cardiovascular disease. The goal is to contribute to the rationale for using generic drugs.

## 2. Methods

### 2.1 Design

A systematic review incorporating meta-analyses was conducted using methods outlined in The Cochrane Handbook for Systematic Reviews of Interventions [16]. This protocol has been reported according to the Preferred Reporting Items for Systematic Reviews and Meta-Analyses (PRISMA) statements [17]. This study has been registered in the International Prospective Register of Systematic Reviews with the registration number CRD42023481597, https://www.crd.york.ac.uk/PROSPERO/view/CRD42023481597.

### 2.2 Sources and Search Strategy 

The search was conducted online using PubMed, Embase, The Cochrane Library, and clinicaltrials.gov databases from inception until December 2023. The search criteria were appropriately adjusted for different databases without altering the overall search strategy. The search strategy refers to the study by Manzoli *et al*. [8] and requires that at least one of the following items be mentioned. (1) Terms related to the study, which include clinical studies, cohorts, and crossover and randomized trials. (2) Terms related to the origin of the drug, including original drugs, brand-name drugs, innovator drugs, patented drugs, generic drugs, non-brand drugs, off-patent drugs, and other brands. (3) Terms related to cardiovascular disease: coronary heart disease, ischemic heart disease, acute coronary syndrome, myocardial infarction, angina pectoris, atrial fibrillation, atrial flutter, heart failure, congestive heart disease, hypertension, hypercholesterolemia, and atherosclerosis. (4) Terms related to medication: angiotensin-converting enzyme inhibitors, angiotensin receptor blockers, antihypertensive drugs, beta-blockers, calcium channel blockers, antithrombotic drugs, antiplatelet drugs, anticoagulants, diuretics, and statins. The articles obtained from the search were required to have complete titles and clear abstracts. Articles from various databases were summarized, and the eligible articles were screened and merged. The search strategy is detailed in **Supplementary File 1**.

### 2.3 Eligibility Criteria

To ensure the accuracy of the literature screening, at least two reviewers performed the screening independently. The following literature was excluded: (1) duplicate publications; (2) incomplete essential information; (3) data that could not be accurately extracted; (4) brand-name drugs were not involved; (5) data lacking outcomes or validation; (6) animal research; (7) research on biological products. The title and abstract were browsed, and the full text was thoroughly read after the irrelevant and repetitive literature had been excluded. Disagreements were resolved through negotiation, and if no consensus could be reached, a third reviewer made the final judgment.

### 2.4 Outcomes Measurement 

Clinical efficacy outcomes included vital signs such as blood pressure (BP), platelet aggregation inhibition (PAI), international normalized ratio (INR), low-density lipoprotein (LDL), and urinary sodium levels. Clinical safety outcomes included major adverse cardiovascular events (MACEs) and adverse events (AEs). MACEs are defined as those that relate to ischemic cardiovascular events such as acute coronary syndrome, myocardial infarction, stroke, thrombosis, and death. AEs are those that occurred during the study, including non-fatal bleeding, hypotension, abdominal pain, diarrhea, allergies, and other events that occurred in subjects after administration of the drug.

### 2.5 Data Extraction

The information was extracted and recorded in Microsoft Excel (Version 2016, Microsoft Corporation, Redmond, Washington, USA) and then cross-checked by two reviewers. The following data were extracted: title, authors, publication date, sample size, inclusion criteria, outcomes, study methodology, risk of bias, categorical variables, results for continuous variables, and other relevant information, such as drug type, age of study subjects, study location, follow-up duration, funding source, protocol registration, and ethical review status. Study authors were contacted as necessary if there was uncertainty in the data or the results needed to be clarified.

### 2.6 Risk of Bias Assessment

The included studies were assessed for bias by two independent reviewers. The Cochrane Risk of Bias Assessment Tool [18] was utilized for RCTs. The Cochrane Risk of Bias Assessment Tool is one of the most comprehensive approaches to assessing the potential for bias in RCTs included in systematic reviews or meta-analyses. The following dimensions were assessed: randomization method, allocation concealment, blinding, completeness of results, selective reporting, and other sources of bias. For the items mentioned above, the included studies were assessed as “Yes” (low risk of bias), “No” (high risk of bias), or “Unclear” (uncertainty or lack of information about the bias situation). Non-randomized controlled trials (non-RCTs) were assessed using a new tool called the ROBINS-I scale [19]. ROBINS-I is used to evaluate the risk of bias and estimates the comparative effectiveness of interventions from studies that did not use randomization to allocate units to comparison groups. The tool will be particularly useful to those undertaking systematic reviews that include non-randomized studies. The ROBINS-I scale consists of seven assessment domains, including confounding, selection bias, bias in measurement and classification of interventions, bias due to deviations from intended interventions, bias due to missing data, bias in measurement of outcomes, and bias in the selection of the reported results. The risk level of the study was thoroughly evaluated based on the risk assessment criteria. The results were classified as low risk, moderate risk, serious risk, critical risk, and no information.

### 2.7 Data Synthesis and Statistical Analysis

Relevant study methodology and clinical characteristics are presented in a preliminary summary. A meta-analysis was conducted using the Cochrane Collaboration’s Review Manager Software (Version 5.4, The Cochrane Collaboration, Nordic Cochrane Centre, Copenhagen, Denmark). The relative risk (RR) ratio was used as the effect analysis statistic for categorical variables. The mean difference was used as the effect analysis statistic for continuous variables, and statistical significance was determined based on the 95% confidence intervals. Dichotomous data are expressed as the RR ratio with 95% confidence intervals, and continuous outcomes are presented as the mean difference (MD) with 95% confidence intervals. Heterogeneity tests of studies were quantified using I^2^, and the magnitude of heterogeneity is expressed as a percentage. The I^2^ statistic describes the percentage of variation between studies (variation not due to sampling error) and the total variation. When studies exhibit high heterogeneity (I^2^ >50%), meta-analyses are performed using a random-effects model; otherwise, a fixed-effect model is adopted [20]. The risk of publication bias assessment between studies is presented through funnel plots [21, 22]. Subgroup analyses and/or meta-regression were conducted to evaluate the influence of sources of heterogeneity based on the following factors: drug classification, study site, study design, follow-up period, and source of grant funding.

Research results from multiple centers worldwide were fully incorporated into the study to guarantee the breadth and quality of the included studies. Regarding regional differences, the possible differences caused by the distribution of subjects in different regions, including Asia, Europe, America, and other areas, were considered, and subgroup analysis was conducted for regional factors. In terms of research funding sources, although not all research is funded, the funded research defines the ways and types of funding sources, including that funded by manufacturers, academic organizations, government, and other foundations, and research with no funding or unknown funding sources, ealongside fully considering the impact of the manufacturer funding the research has on the results. In terms of research design, the included studies were divided into two categories, focusing on whether the study was an randomized controlled trial (RCT) and observing the impact of the study design on the research results. In terms of the study follow-up time, subgroup analysis was conducted for studies with a follow-up time ≤30 days and studies with a follow-up time >30 days to explore whether the follow-up time could significantly impact the results. This study conducted a comprehensive subgroup analysis of the time, region, funding source, and research type to ensure accurate and reliable research results.

## 3. Results 

### 3.1 Characteristics of Studies 

The initial search yielded 4238 relevant papers. After eliminating duplicates, 132 were screened according to the inclusion criteria, and 45 papers were subsequently excluded. Among the excluded papers, four studies did not mention the brand name drug, enine switched to generic treatment midway through the study, and 32 could not be extracted due to incomplete data. A total of 87 papers were included in the qualitative analyses, and data were validly extracted from studies; 57 papers were included in the quantitative analyses. MACEs were extracted from 19 studies (n = 384,640). Additionally, 35 studies reported other adverse events (n = 580,125), and 27 addressed at least one clinical effectiveness outcome (n = 16,737). All included studies reported on differences between brand-name and generic drugs. The detailed literature screening process is documented in **Supplementary File 1**.

**Fig. 1.**
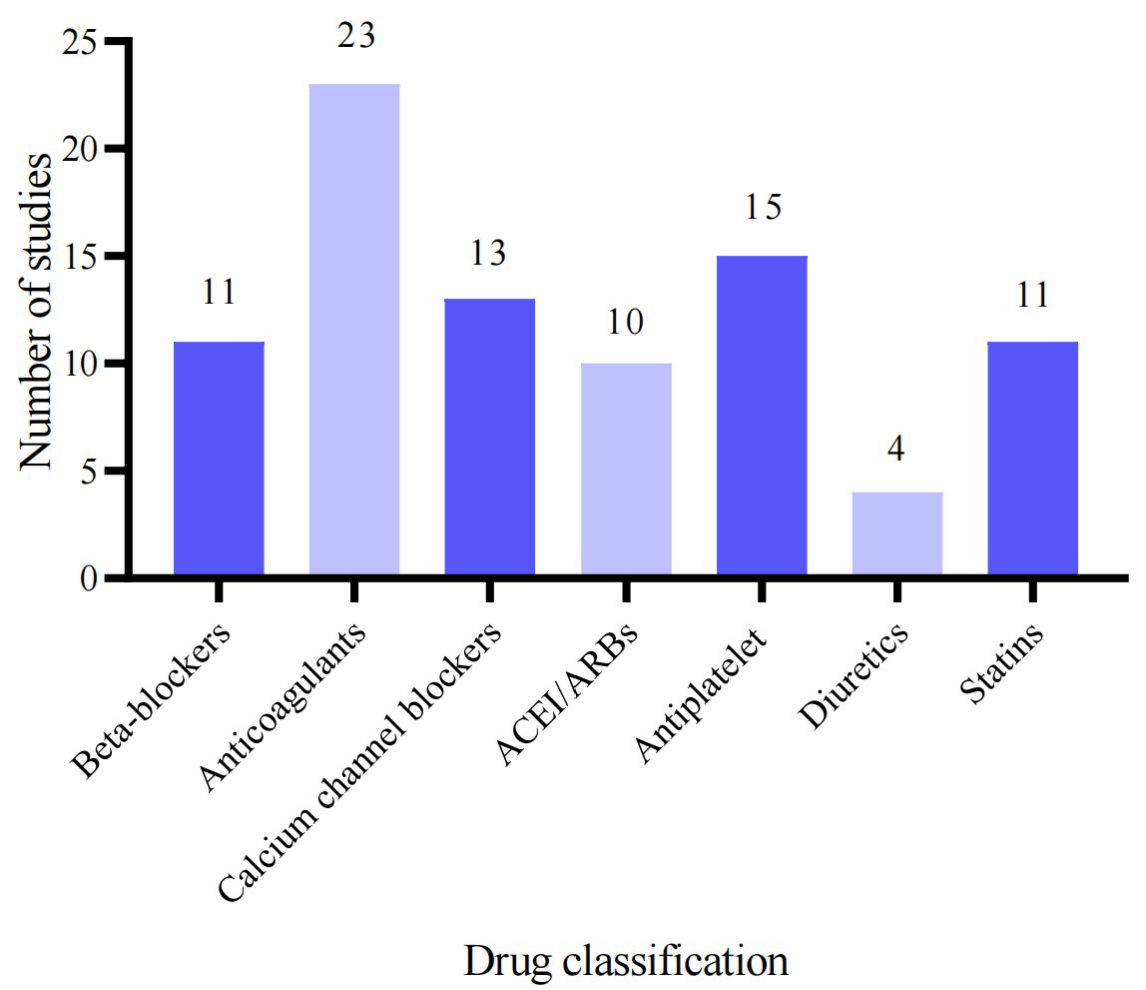
**Drug classification statistics included in the study**. ACEI, angiotensin-converting enzyme inhibitor; ARBs, angiotensin receptor blockers.

In the preliminary qualitative study, more than 2 million subjects were enrolled in the use of cardiovascular drugs, such as angiotensin receptor blockers (ARBs), angiotensin-converting enzyme inhibitors (ACEIs), β-blockers, calcium channel blockers, antiplatelet agents, anticoagulants, diuretics, statins, and other related therapeutic agents (Fig. 1). Since the 1980s, there has been a growing number of studies related to the rise in the use of generics, showing a clear upward trend in the number of studies over the decades. The production of generic drugs is a global industry, with associated studies being conducted worldwide. There were 38 relevant studies published in Asia, 19 in Europe, 27 in the Americas, and 3 in other regions. There were 56 RCTs, accounting for 64.37%, and 31 non-randomized clinical trials, including crossover and parallel trials and clinical observational studies. The follow-up period ranged from 1 day to 7 years, with 41 studies having a more than 30 days follow-up period. A total of 58 studies received funding from various sources, with 41 studies funded and supported by drug manufacturers or pharmaceutical companies. Only 15 studies were registered online and received a valid protocol registration number, while 79 studies were reviewed and approved by the ethics committee. The basic characteristics of the included studies are presented in Table 1 (Ref. [5, 6, 15, 23, 24, 25, 26, 27, 28, 29, 30, 31, 32, 33, 34, 35, 36, 37, 38, 39, 40, 41, 42, 43, 44, 45, 46, 47, 48, 49, 50, 51, 52, 53, 54, 55, 56, 57, 58, 59, 60, 61, 62, 63, 64, 65, 66, 67, 68, 69, 70, 71, 72, 73, 74, 75, 76, 77, 78, 79, 80, 81, 82, 83, 84, 85, 86, 87, 88, 89, 90, 91, 92, 93, 94, 95, 96, 97, 98, 99, 100, 101, 102, 103, 104]).

**Table 1.** **Basic characteristics of included studies**.

Author/Year	Region	Drugs	RCT	Population	Sample size	Age	Follow-up	Outcome	Funding	Registration
ACEI/ARBs										
Portolés A *et al*., 2004 [23]	Spain	Enalapril	Yes	Healthy	23	23	36 h	BP, MACE, AE	No	No
Kim SH *et al*., 2009 [24]	Korea	Ramipril	Yes	HTN	89	50	8 w	BP, MACE, AE	MFGR	No
Spínola ACF *et al*., 2009 [25]	Canada	Valsartan	Yes	Healthy	41	37	36 h	MACE, AE	MFGR	No
Iqbal M *et al*., 2010 [26]	India	Valsartan	No	Healthy	18	25	24 h	AE	MFGR	No
Jia JY *et al*., 2010 [27]	China	Losartan	Yes	Healthy	27	24	36 h	BP, HR, MACE, AE	MFGR	No
Li KY *et al*., 2010 [28]	China	Olmesartan	No	Healthy	21	21	48 h	AE	MFGR	2005L01077
Oigman W *et al*., 2013 [29]	Brazil	Ramipril	Yes	HTN	102	57	8 w	BP, MACE, AE	MFGR	ISRCTN05051235
Leclerc J *et al*., 2017 [6]	Canada	Sartans	No	HTN	136,177	76	1095 d	MACE	No	No
Huang T *et al*., 2022 [30]	China	Sartans	Yes	HTN	8808	59	90 d	MACE	No	No
Patel R *et al*., 2017 [31]	India	Candesartan	No	Healthy	18	30	21 d	BP, AE	MFGR	NCT0002254447
Anticoagulants										
Weibert RT *et al*., 2000 [32]	US	Warfarin	Yes	AF	104	70	4 w	INR, MACE, AE	MFGR	No
Lee HL *et al*., 2005 [33]	Taiwan	Warfarin	Yes	Valve surgery	35	52	12 w	INR, AE	MFGR	No
Pereira JA *et al*., 2005 [34]	Canada	Warfarin	No	AF or DVT	7	63	15 w	INR	No	No
Kwong WJ *et al*., 2012 [35]	US	Warfarin	Yes	AF	12,908	67	365 d	MACE	MFGR	No
Hellfritzsch M *et al*., 2016 [36]	Danish	Warfarin	Yes	AF, VTE, valve surgery	105,751	72	660 d	INR, MACE, AE	No	No
Leclerc J *et al*., 2019 [5]	Canada	Warfarin	No	CVD	280,158	58	7300 d	MACE	No	No
Gomes M *et al*., 2011 [37]	Brazil	Enoxaparin	Yes	VTE prevention	200	50	60 d	MACE, AE	Private	No
Grampp G *et al*., 2015 [38]	US	Enoxaparin	Yes	DVT, PE, ACS	218,566	N/A	180 d	AE	No	No
Ramacciotti E *et al*., 2018 [39]	Brazil	Enoxaparin	Yes	VTE	243	52	64 d	MACE, AE	MFGR	No
Abdolvand M *et al*., 2019 [40]	Iran	Enoxaparin	Yes	VTE	220	38	10 d	MACE, AE	No	IRCT20090914002459N2
Casella IB and Puech-Leão P, 2015 [41]	Brazil	Enoxaparin	Yes	Prevention of DVT and VTE	114	67	7 d	MACE, AE	Private	No
Desai RJ *et al*., 2020 [42]	US	Warfarin	Yes	Anticoagulant	33,645	77	365 d	MACE, AE	FDA	No
Fantoni C *et al*., 2021 [43]	Italy	Enoxaparin	No	Abdominal surgery	381	69	N/A	MACE, AE	No	No
Gomes Freitas C *et al*., 2021 [44]	Brazil	Warfarin	Yes	AF and/or AFL	17	68	4 w	INR	Academia	No
Feng L *et al*., 2009 [45]	China	Enoxaparin	No	Healthy	22	21	24 h	AE	No	No
Antiplatelet										
Rao TRK *et al*., 2003 [46]	India	Clopidogrel	Yes	Healthy	20	27	10 d	PAI, MACE, AE	MFGR	No
Kim SD *et al*., 2009 [47]	Korea	Clopidogrel	Yes	Healthy	44	24	13 d	PAI, MACE, AE	MFGR	No
Di Girolamo G *et al*., 2010 [48]	Argentina	Clopidogrel	No	Healthy	24	34	12 h	AE	MFGR	No
Müller A *et al*., 2010 [49]	Venezuela	Clopidogrel	Yes	Healthy	20	23	7 d	PAI	No	No
Shim CY *et al*., 2010 [50]	Korea	Clopidogrel	Yes	Healthy	29	29	1 w	PAI, AE	MFGR	No
Khosravi AR *et al*., 2011 [51]	Iran	Clopidogrel	No	PCI	442	59	6 m	MACE, AE	MFGR	IRCT138712111723N1
Suh JW *et al*., 2011 [52]	Korea	Clopidogrel	Yes	CVD	203	62	4 w	MACE, AE	MFGR	NCT00947843
Oberhänsli M *et al*., 2012 [53]	Swiss	Clopidogrel	Yes	CVD	60	69	10 d	PAI, AE	Academia	No
Tsoumani ME *et al*., 2012 [54]	Greece	Clopidogrel	No	ACS	86	70	6 m	Platelet reactivity index	MFGR	No
Tsoumani ME *et al*., 2012 [55]	Greece	Clopidogrel	No	ACS	96	64	4 w	PAI	Academia	No
Park JB *et al*., 2013 [56]	Korea	Clopidogrel	Yes	CVD	130	62	4 w	PAI, MACE, AE	MFGR	NCT01584791
Komosa A *et al*., 2015 [57]	Poland	Clopidogrel	No	CVD	53	49	8 d	PAI, MACE	No	No
Seo KW *et al*., 2014 [58]	Korea	Clopidogrel	No	ACS	95	58	4 w	PAI, MACE, AE	MFGR	NCT02060786
Park YM *et al*., 2012 [59]	Korea	Clopidogrel	Yes	CAD, DES	428	62	365 d	MACE	No	No
Kovacic JC * et al*., 2014 [60]	Canada	Clopidogrel	No	PCI	11,284	65	30 d	MACE	MFGR	No
Hamilos M *et al*., 2015 [61]	NA	Clopidogrel	No	CAD	101	64	14 h	PAI	MFGR	No
Ntalas IV * et al*., 2016 [62]	Greece	Clopidogrel	Yes	CAD	1557	70	365 d	MACE	MFGR	NCT02126982
Hajizadeh R *et al*., 2017 [63]	Iran	Clopidogrel	Yes	PCI	129	58	30 d	PAI	Academia	No
Westphal ES *et al*., 2022 [15]	NA	Clopidogrel	Yes	Stroke	439	N/A	14 d	MACE, AE	Private	No
Ko DT *et al*., 2018 [64]	Canada	Clopidogrel	Yes	ACS	24,530	77	365 d	MACE	Private	No
Leclerc J *et al*., 2019 [65]	Canada	Clopidogrel	No	CVD	89,525	78	1095 d	MACE	No	No
Patsourakos NG *et al*., 2020 [66]	Greece	Clopidogrel	No	ACS	1194	65	365 d	MACE	MFGR	No
Zarif B *et al*., 2022 [67]	Egypt	Ticagrelor	Yes	Healthy	33	38	4 d	PAI, MACE, AE	Private	No
Beta-blockers										
Carter BL *et al*., 1989 [68]	USA	Propranolol	Yes	HTN	12	46	4 w	BP	Academia	No
el-Sayed MS and Davies B, 1989 [69]	UK	Propranolol	Yes	Healthy	12	20	2 h	BP	No	No
Sarkar MA *et al*., 1995 [70]	USA	Atenolol	No	Healthy	29	N/A	24 h	BP, HR, AE	MFGR	No
Cuadrado A *et al*., 2002 [71]	Spain	Atenolol	Yes	Healthy	24	23	30 h	BP, MACE, AE	MFGR	No
Portolés A *et al*., 2005 [72]	Spain	Carvedilol	No	Healthy	24	23	24 h	AE	No	No
Liu Y *et al*., 2013 [73]	China	Carvedilol	Yes	Healthy	23	27	24 h	MACE, AE	MFGR	No
Ahrens W *et al*., 2007 [74]	Germany	Metoprolol	No	CVD	49,673	56	193 d	MACE, AE	MFGR	No
Chanchai R *et al*., 2018 [75]	Thailand	Carvedilol, Bisoprolol	Yes	HF	217	58	168 d	BP, AE	Academia	No
Huang T *et al*., 2022 [30]	China	Metoprolol	Yes	HTN	2526	60	90 d	MACE	No	No
Aretha D *et al*., 2020 [76]	Greece	Esmolol	Yes	SVT and HTN	31	74	24 h	BP, HR	No	No
Mosley SA *et al*., 2022 [77]	US	Metoprolol	No	HTN	36	53	28 d	BP, HR	FDA	No
Calcium channel blockers										
Rani Usha P *et al*., 1997 [78]	India	Diltiazem	No	Healthy	12	27	12 h	BP	MFGR	No
Saseen JJ * et al*., 1997 [79]	US	Verapamil	No	HTN	8	70	2 w	BP, AE	No	No
Park JY *et al*., 2004 [80]	Korea	Amlodipine	No	Healthy	18	22	6 d	BP, AE	No	No
Kim SH *et al*., 2007 [81]	Korea	Amlodipine	Yes	HTN	188	53	8 w	BP, MACE, AE	MFGR	No
Mignini F *et al*., 2007 [82]	Italy	Amlodipine	Yes	Healthy	24	35	6 d	BP, MACE, AE	No	No
Kim SA *et al*., 2008 [83]	Korea	Amlodipine	Yes	HTN	124	53	8 w	BP, MACE, AE	No	No
Liu Y *et al*., 2009 [84]	China	Amlodipine	Yes	Healthy	20	21	5 d	MACE, AE	Academia	No
Pollak PT * et al*., 2017 [85]	Canada	Nifedipine	Yes	Healthy	20	64	14 d	BP	MFGR	No
Desai RJ *et al*., 2019 [86]	US	Amlodipine	No	HTN	1,069,796	55	365 d	MACE	FDA	No
Huang T *et al*., 2022 [30]	China	Dipines	Yes	HTN	9736	63	90 d	MACE	No	No
Tung YC * et al*., 2020 [87]	China	Nifedipine	Yes	HTN	98,335	N/A	1460 d	MACE, AE	No	No
Lee HW *et al*., 2022 [88]	China	Nifedipine	Yes	HTN	5970	69	900 d	MACE	MFGR	No
Tung YC *et al*., 2022 [89]	China	Nifedipine	Yes	HTN	4204	63	90 d	MACE	No	No
Diuretics										
Martin BK *et al*., 1984 [90]	UK	Furosemide	No	Healthy	12	30	24 h	Urine sodium	Academia	No
Pan HY *et al*., 1984 [91]	Hong Kong	Furosemide	Yes	HF	5	N/A	8 h	Urine sodium	No	No
Murray MD * et al*., 1997 [92]	US	Furosemide	Yes	HTN or HF	17	65	2 w	Urine sodium	MFGR (Brand)	No
Almeida S *et al*., 2011 [93]	Portugal	Eplerenone	Yes	Healthy	27	40	24 h	MACE, AE	MFGR	No
Statins										
Wiwanitkit V *et al*., 2002 [94]	Thailand	Simvastatin	Yes	Healthy	37	49	16 w	LDL, AE	MFGR	No
Kim SH *et al*., 2010 [95]	Korea	Atorvastatin	Yes	CVD	235	61	8 w	LDL, MACE, AE	MFGR	NCT01029522
Liu YM *et al*., 2010 [96]	China	Atorvastatin	No	Healthy	45	24	48 h	AE	MFGR (Brand)	CNR2007L02512
Kim SH *et al*., 2013 [97]	Korea	Atorvastatin	Yes	Hypercholest.	289	61	8 w	LDL, MACE, AE	MFGR	NCT01285544
Corrao G *et al*., 2014 [98]	Italy	Simvastatin	No	CVD	13,799	63	1278 d	MACE	Academia	No
Gagne JJ *et al*., 2014 [99]	US	Statins	No	CVD	90,111	76	365 d	MACE	MFGR	No
Jackevicius CA *et al*., 2016 [100]	Canada	Statins	Yes	ACS	15,726	77	365 d	MACE	Private	No
Lee JH *et al*., 2017 [101]	Korea	Atorvastatin	Yes	Hypercholest.	346	63	56 d	LDL	MFGR	No
Sicras-Mainar A *et al*., 2018 [102]	Spain	Statins	Yes	Hypercholest.	13,244	61	1825 d	LDL, MACE	MFGR	No
Kim H *et al*., 2020 [103]	Korea	Rosuvastatin	Yes	Lipid-lowering	158	60	12 w	LDL, MACE, AE	No	NCT03949374
Manasirisuk P *et al*., 2021 [104]	Thailand	Atorvastatin	Yes	CVD	488	61	270 d	LDL, AE	No	No

HTN, hypertension; AF, atrial fibrillation; DVT, deep vein thrombosis; VTE, venous thrombosis embolism; PE, pulmonary thromboembolism; PCI, percutaneous coronary intervention; CVD, cardiovascular disease; DES, drug-eluting stent; CAD, coronary artery disease; HF, heart failure; SVT, supraventricular tachycardia; hypercholest, hypercholesterolemia; N/A, not applicable; h, hour; d, day; w, week; y, year; BP, blood pressure; HR, heart rate; MACE, major adverse cardiovascular event; AE, adverse event; LDL, low-density lipoprotein; PAI, platelet aggregation inhibition; MFGR, manufacturer; RCT, randomized controlled trial; INR, international normalized ratio; ACS, acute coronary syndrome; FDA, Food and Drug Administration; AFL, atrial flutter.

### 3.2 Meta-Analysis

Of all the included studies, 71 showed no significant difference between generic and brand-name drugs. Out of 20 studies, a significant difference was found between the two types, with 15 of these showing better clinical efficacy and safety after using the brand-name drug. Additionally, five studies concluded that generic drugs are more effective than brand-name drugs.

**Fig. 2.**
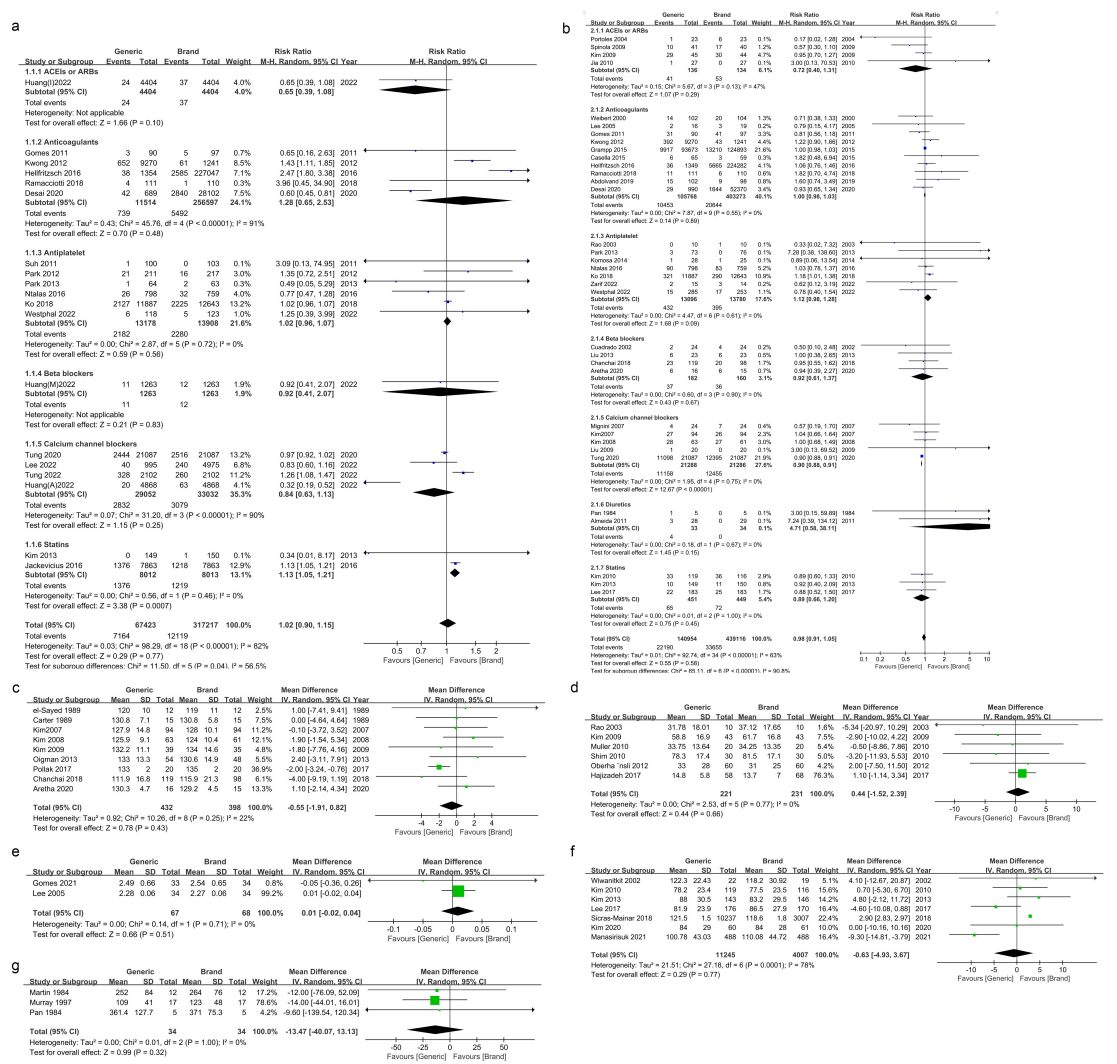
**Meta-analysis of clinical efficacy and safety of branded versus generic drugs for the treatment of cardiovascular disease**. (a) MACEs. (b) AEs. (c) BP. (d) PAI. (e) INR. (f) LDL. (g) Urinary sodium. M-H, mantel-haenszel; IV, inverse variance.

Regarding safety, 19 studies were included to assess MACEs, with a high overall heterogeneity of studies (I^2^: 82%). Random-effects model analysis showed that the overall risk of MACEs was comparable for generic versus brand-name drugs (RR 1.02 [0.90–1.15]) (Fig. 2a). For cardiovascular medications other than statins, the risk ratios of ACEI/ARB (RR 0.65 [0.39, 1.08]), anticoagulants (RR 1.28 [0.65, 2.53]), antiplatelet agents (RR 1.02 [0.96, 1.07]), beta-blockers (RR 0.92 [0.41, 2.07]), and calcium channel blockers (RR 0.84 [0.63, 1.13]) for MACEs were not statistically different. Conversely, statins performed differently from the above drugs, and pooled analyses revealed a relatively higher risk of MACEs with generic statins (RR 1.13 [1.05, 1.12]). Furthermore, AEs were effectively extracted from 36 studies, and statistical heterogeneity was found across studies (I^2^: 62%). The risk of AEs was similar (RR 0.98 [0.91–1.05]) for generic versus brand-name drugs (Fig. 2b). Further analyses showed a statistically significant risk of AEs with calcium channel blockers, with a more prominent overall effect from generics (RR 0.90 [0.88, 0.91]). In addition, ACEIs/ARBs (RR 0.72 [0.40, 1.31]), anticoagulants (RR 1.00 [0.98, 1.03]), antiplatelet agents (RR 1.12 [0.98, 1.28]), beta-blockers (RR 0.92 [0.61, 1,37]), diuretics (RR 4.71 [0.58, 38.11]), statins (RR 0.89 [0.66, 1.20]), and other drugs (RR 0.98 [0.91, 1.05]) showed no statistically significant difference in the risk of adverse events.

Regarding efficacy, the following data were extracted based on available vital signs and hospital laboratory test results: BP, PAI, INR, LDL, and urinary sodium levels. There was high heterogeneity in the LDL-related studies (I^2^: 78%), with no obvious heterogeneity observed in the remaining studies (Fig. 2). Mean BP values were extracted after subjects received administration of drugs between the two groups from nine studies, and systolic blood pressure was chosen as the index of evaluation; the drugs included in the studies were ACEI/ARB, β-blockers, and calcium channel blockers (Fig. 2c). PAI was extracted from seven studies related to antiplatelet drugs (Fig. 2d). INR was extracted from two studies on anticoagulants (Fig. 2e). LDL was extracted from three studies associated with lipid-lowering drugs (Fig. 2f). Data on urinary sodium levels were extracted from three studies related to diuretics (Fig. 2g). The comparisons indicated that the risk ratios for the above drugs fluctuated within a range, but no statistically significant difference in effect was observed between generic and brand-name drugs.

### 3.3 Subgroup Analysis

Subgroup analyses were conducted for a variety of different factors; MACEs were compared between the two groups: (1) region: studies in Asia (0.86 [0.68, 1.09]), Europe (1.40 [0.44, 4.49]), America (0.99 [0.49, 2.02]), other regions (1.25 [0.39, 3.99]); (2) study design: RCTs (0.81 [0.52, 1.27]) vs. non-RCTs (1.03 [0.91, 1.17]); (3) follow-up time: studies with ≤30 days of follow-up (1.16 [0.43, 3.12]) vs. studies with >30 days of follow-up (1.02 [0.90, 1.15]); (4) sources of funding: manufacturer-funded studies (1.03 [0.71, 1.49]), academic organizations, government and other foundation funding (0.97 [0.83, 1.13]), and studies with no funding or unknown funding sources (1.01 [0.73, 1.38]).

AEs were compared between the two groups: (1) region: studies in Asia (0.90 [0.88, 0.91]), Europe (1.00 [0.82, 1.22]), America (1.02 [0.91, 1.15]), other regions (0.76 [0.41, 1.41]); (2) study design: RCTs (0.94 [0.83, 1.06]) vs. non-RCTs (1.00 [0.91, 1.10]); (3) follow-up time: studies with ≤30 days of follow-up (0.85 [0.65, 1.10]) vs. studies with >30 days of follow-up (0.99 [0.92, 1.07]); (4) sources of funding: manufacturer-funded studies (0.99 [0.86, 1.13]), academic organizations, government and other foundation funding (1.07 [0.94, 1.21]), and studies with no funding or unknown funding sources (0.96 [0.87, 1.06]).

We discovered that brand-name cardiovascular drugs in Asia had a higher risk of AEs than generic drugs; meanwhile, there was no statistical difference in risk between generic and brand-name drugs in the remaining subgroup analyses. Overall, study design, follow-up duration, and funding did not affect the risk of MACEs and AEs. Unfortunately, the limited number of studies that included subgroups could not support more detailed analyses (**Supplementary Fig. 1**).

### 3.4 Risk of Bias Assessment 

A total of 57 RCTs were included, of which the randomization method process was mentioned and described in 35, while 21 studies only referred to sample randomization without providing a detailed description, the one remaining study lacked the information to judge. Twelve studies described allocation concealment, and 16 provided details about eimplementing blinding. Twelve studies were designed as double-anonymized. There were different levels of bias comprising three areas: completeness of outcome data, selective reporting, and other sources of bias. In the non-randomized clinical studies, the inclusion of various studies presented different risks of bias. Low and moderate risks were identified in the confounding bias and bias in selecting the reported result entries. Serious selection bias was noted in two studies, while two studies contained serious bias in the measurement classification of intervention risk. One study found serious bias due to deviations from the intended interventions, and another study possessed serious bias due to the risk of missing data. Finally, one study presented that serious bias resulted from the measurement of outcomes. A few studies did not present any available information pertaining to the items mentioned above (**Supplementary Table 1**).

### 3.5 Publication Bias

A funnel plot was performed for the included studies, all of which were full-text studies. The plot exhibited a largely symmetrical scatter distribution on both sides and a dispersed distribution of study intervals. There was no significant publication bias (**Supplementary Fig. 3**).

## 4. Discussion

Generic medicines play a key role in healthcare expenditure, costing on average 30% less than brand-name drugs [105]. Doctors, pharmacists, and drug users have expressed distrust and uncertainty regarding the safety and efficacy of generic drugs [12, 13]. Meanwhile, the availability of generic alternatives often complicates drug adherence, and a significant number of patients hold a negative perception of generics [106].

Since the 1980s, bioequivalence trials have increased, leading to a wealth of clinical findings. Comparatively, Flacco *et al*. [107] recently conducted a study in which they gathered 186 completed trials comparing the safety and efficacy of brand-name and generic drugs. Flacco and co-authors [107] extracted data from 93 trials, almost all of which reported positive results. The results favored generic medications, but the literature generally had a high risk of bias. Manzoli *et al*. [8] summarized 74 randomized controlled trials evaluating soft outcomes such as BP and LDL levels and MACEs, and the conclusions supported the clinical equivalence of brand-name and generic drugs. However, the previous sources of evidence were not ideal, with cross-design studies accounting for 78.37% and bioequivalence studies accounting for 56.75%. The research focused on relative equivalence and pharmacokinetic characteristics. Additionally, the sample size was small, meaning the hard outcomes that can be extracted are limited, and the conclusions still need to be verified. In addition, Leclerc *et al*. [7] questioned the effectiveness and safety of generic drugs used in cardiology. This is the first time in recent years that a disparity in all-cause hospital visits in cardiology has been observed between generic and brand-name drugs. Over half of the 72 studies demonstrated similar effectiveness and safety between generic and brand-name cardiovascular medications [7]. The systematic review included abstract-type articles, making it difficult to ensure the comprehensiveness of information and indirectly introducing multiple confounds. Overall, the available evidence was too varied to conclusively support the claim that generic drugs are as effective as brand-name drugs.

Our study was conducted on drugs commonly used to treat cardiovascular diseases, including a wide range of antihypertensive drugs, antithrombotic drugs, diuretics, and lipid-lowering drugs. Both non-RCTs and randomized controlled studies were included in the study. There were no significant differences in the safety and efficacy of brand-name and generic drugs for treating cardiovascular disease, except for statins and calcium channel blockers. Moreover, the analysis found a significantly higher risk of MACEs with generic statins. Therefore, it was recommended to carefully consider the use of such generic drugs in the course of clinical treatment. For ACEI/ARB, anticoagulant drugs, antiplatelet drugs, β-blockers, and diuretics, the risks of safety and efficacy outcomes of generic drugs and brand drugs are basically similar, and theoretically, they can be used as substitutes for each other. In the subgroup analyses performed in this study, we were particularly interested in the variations in common adverse events associated with cardiovascular drugs in different regions. Compared to Europe, the Americas, and other regions, we found that branded drugs in Asia had a significantly higher risk of AEs than generic drugs, contrasting with previous findings [7]. A related study has examined whether generic medications do not compromise therapeutic benefits and may improve patient compliance [108]. However, no prior study has definitively concluded that there is a shortage of brand-name medications. Overall, variations in drug use across different regions should be interpreted with caution and may be associated with factors such as racial disparities among subjects from different areas and patterns of reporting adverse reactions [109]. At the same time, numerous evaluations of generic drugs are currently being conducted in Asia, involving a wide and diverse range of drug sources. The ongoing drug evaluations must be rigorous, making it challenging to draw premature conclusions. Therefore, it is essential to establish a robust evaluation system and measurement criteria to ensure reliable data validate the current results.

This review provides a detailed overview of study locations, timing, sources, grant funding, and registrations. The earliest eligible published study is from 1984; thus, studies spanning nearly three decades have been included, covering a wide range of cardiovascular drug studies. The study area covers a broad geographical area, including Asia, Europe, America, and other regions. Many bioequivalences and clinical observational studies were included regarding study design and subjects. Additionally, real-world data studies provided reliable evidence for this analysis, allowing for more diverse data extraction. The follow-up period was extended compared to previous studies, encompassing both short- and long-term observations or follow-up periods, and the data were more comprehensive. Subgroup analysis was performed to explore possible drug variations while accounting for multiple factors, resulting in clear and extensive research.

The following study limitations require attention: Firstly, heterogeneity among the included studies was analyzed using a random-effects model. However, there is objective heterogeneity in the studies, which may impact the determination of the findings. Secondly, the quality of evidence analysis may be influenced by confounding factors. The data available from accessible studies are limited for conducting further subgroup analyses to examine differences in the gender, age, and ethnicity of the subjects. In the classification of drugs into subgroups, there was a difference in the risk of adverse events between statins and calcium channel blockers when comparing generic drugs to brand-name drugs. Furthermore, the number of drug-related studies was limited due to the diverse sources of the included studies. A rigorous interpretation was conducted to address these aforementioned differences. Additionally, it was not possible to include all relevant studies entirely because of potential publication bias. Only 17.58% of the articles were registered and published on the public network of the study. Some studies were registered, but the results were not published or disclosed in time. As a result, the risk of publication bias could not be eliminated, and the likelihood of biased results being reported and published increased. The delay bias caused by non-publication and delayed publication may overestimate the actual efficacy of generic drugs, impacting individual clinical treatment and health decisions. Finally, the proportion of research funded by the manufacturers of generic drugs was the highest at 45.1%, with only 2.2% of research funding coming from branded drug manufacturers. The research evidence from the government explicitly emphasizes that it was not representative of any opinion or position; however, determining the potential impact of sponsorship bias remains difficult, as no significant differences were observed in the stratified analysis. Effectiveness subgroup analyses of drugs were not performed due to the limited amount of relevant literature that could be included. In addition, a high proportion of crossover design studies were included due to the variable quality of evidence from previous studies. It is challenging to extract meaningful results from the above studies due to the problem of short follow-up periods, while limitations in sample size may restrict the observation of potential outcomes. It is sometimes difficult to conduct randomized studies for ethical, feasibility, and other reasons. Currently, non-randomized studies can supplement RCTs, and the population characteristics are closer to the real world, which is suitable for studying long-term outcome indicators and adverse reactions. Since the interventions were not randomly assigned, the results were more susceptible to various potential biases. However, we used assessment tools to evaluate the risk of bias and more scientifically and carefully screen out high-quality, non-randomized studies.

A remarkable trend exists toward the globalization of generic drugs. The diverse sources of drug manufacturers offer more options for physicians and patients; however, such diversification also comes with the risk of inadequate therapeutic substitution of drugs, whereby drugs use the same generic name but with different trademarks. A generic drug is defined as a product that is marketed by more than one manufacturer and contains the same active pharmaceutical ingredient in the same dosage form, typically referred to as a multi-source drug [110]. The origin of generic ingredients varies worldwide, and there is a lack of standardized control throughout the manufacturing processes. While the consistency evaluations of generic drugs have focused on bioequivalence, the significant challenge lies in determining the clinical equivalence of existing generics. Indeed, the increasing number of generic drugs highlights the inadequacy of evidence based on existing data, emphasizing the need for evidence from large samples and high-quality RCTs. Meanwhile, the results of the trials supported by non-profit funding will be more convincing. The experience implies that local health policies can influence the utilization of a particular generic drug, and the regulation and availability of generic drugs differ from one region to another. As a result, it is challenging to guarantee that generic medications can be completely effective substitutes for brands in clinical settings, as this may require more time to validate and address complex issues. Inadequate evidence is often accompanied by clinical uncertainty; thus, the use of generic drugs should be guided by the opinions of physicians, pharmacists, and other healthcare professionals. The research and evaluations of drugs will continue even after patents expire, meaning comprehensive collaboration among clinical guideline developers, regulatory agencies, policymakers, and the scientific community is necessary to establish drug surveillance strategies and data registries [111]. Meanwhile, improving the construction of the adverse signal reporting system and advancing the quality management of generic drugs are also required alongside the enhancement of safety monitoring mechanisms and the assurance of consistent quality standards for generic drugs. Furthermore, improving the construction of bad signal reporting systems and promoting the quality management of generic drugs to enhance public recognition is necessary. Strengthening safety monitoring mechanisms to ensure consistency in generic drug quality standards is also important.

## 5. Conclusions 

In general, cardiovascular drugs include more types of generic drugs, yet these remain in the minority of the used drugs, even though brand-name drugs have discrepancies. Currently, generic drugs cannot directly and completely replace brand-name drugs in treating cardiovascular diseases.

Given the overall development trend of multi-manufacturer generic drugs in the future, this study provides a strong basis for the global application of generic drugs, clarifying the feasibility of generic drugs in terms of efficacy and safety in cardiovascular diseases. However, some drugs still need to be improved to replace the original drugs in clinical practice. Finally, large-sample, multi-center, high-quality studies remain required to guide the clinical application of cardiovascular drugs and guarantee the safety of medications.

## Availability of Data and Materials

All data points generated or analyzed during this study are included in this article and there are no further underlying data necessary to reproduce the results.

## Supplementary Material

Supplementary material associated with this article can be found, in the online version, at https://doi.org/10.31083/RCM26116.
