# Supplementary material for: Can Generic Medications Be a Safe and Effective Alternative to Brand-Name Drugs for Cardiovascular Disease Treatment? A Systematic Review and Meta-Analysis
Source: Rev Cardiovasc Med. 2025 Mar 7;26(3):26116. doi: 10.31083/RCM26116 (PMC11951291; doi:10.31083/RCM26116)

Supplementary Fig. 1. Subgroup analysis comparing major adverse cardiovascular events (MACE) between the two groups

Supplementary Fig. 1 (A) Subgroup analysis according to region


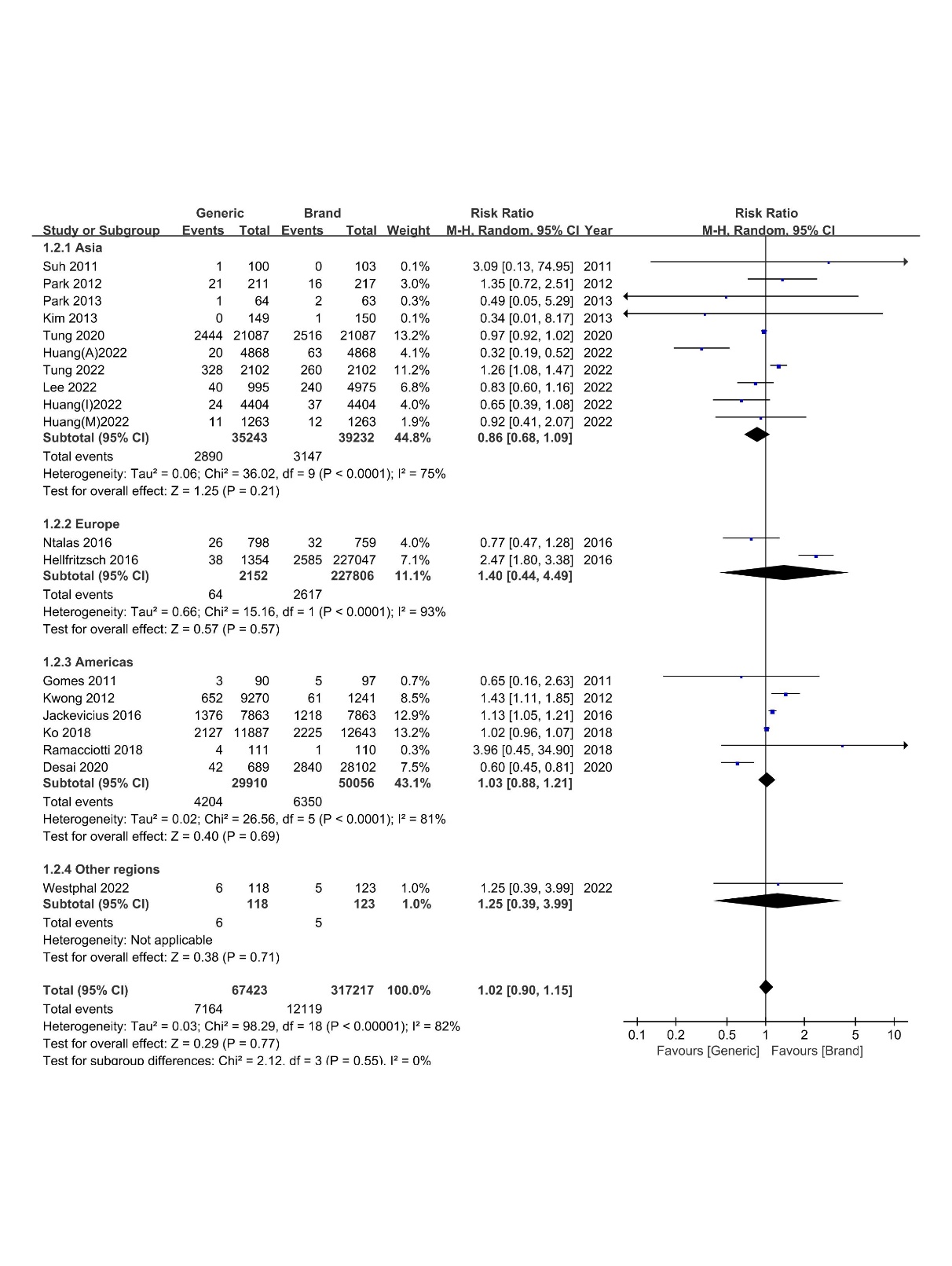


Supplementary Fig. 1 (B) Subgroup analysis according to study design


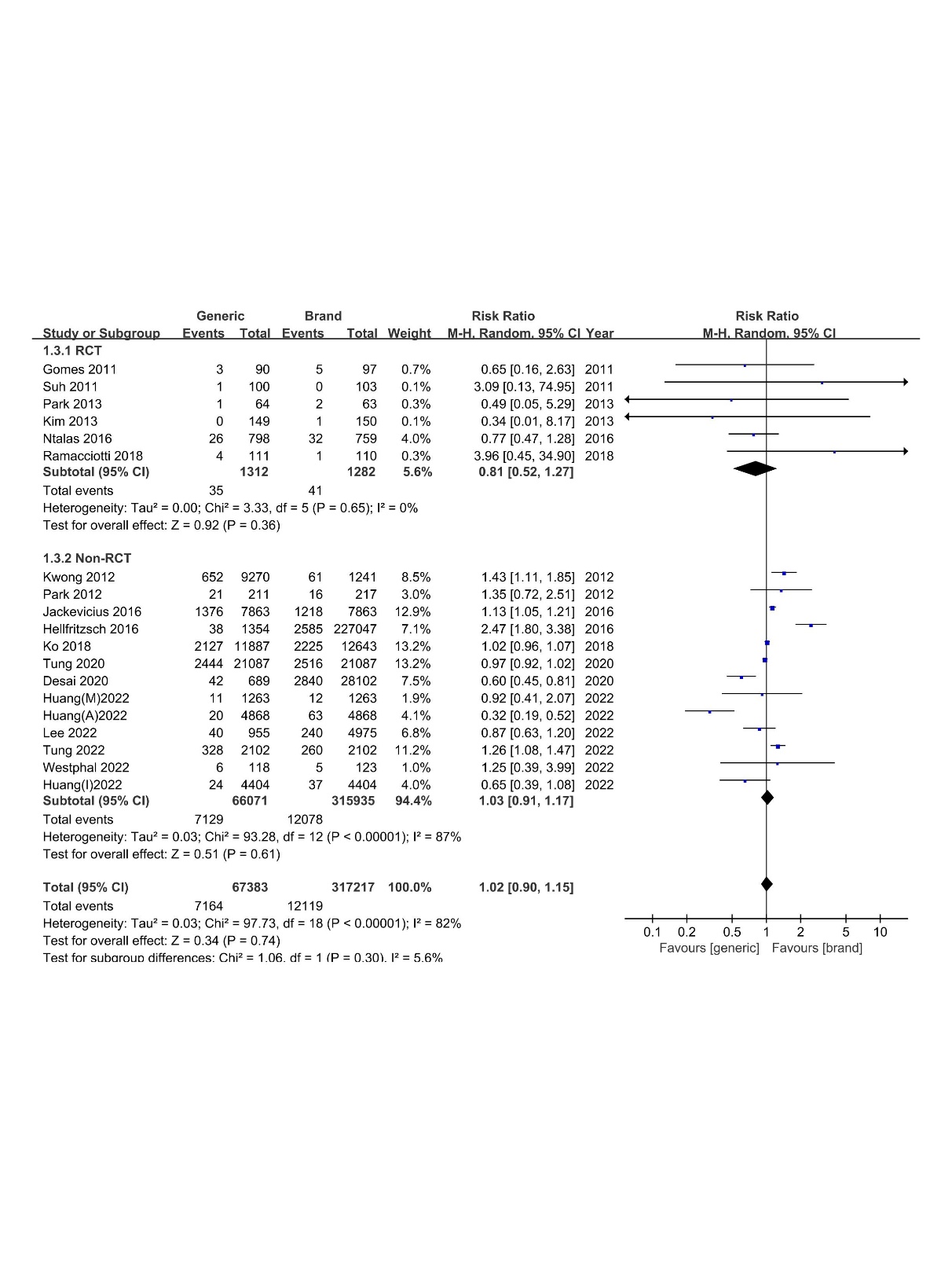


Supplementary Fig. 1 (C) Subgroup analysis according to the follow-up time


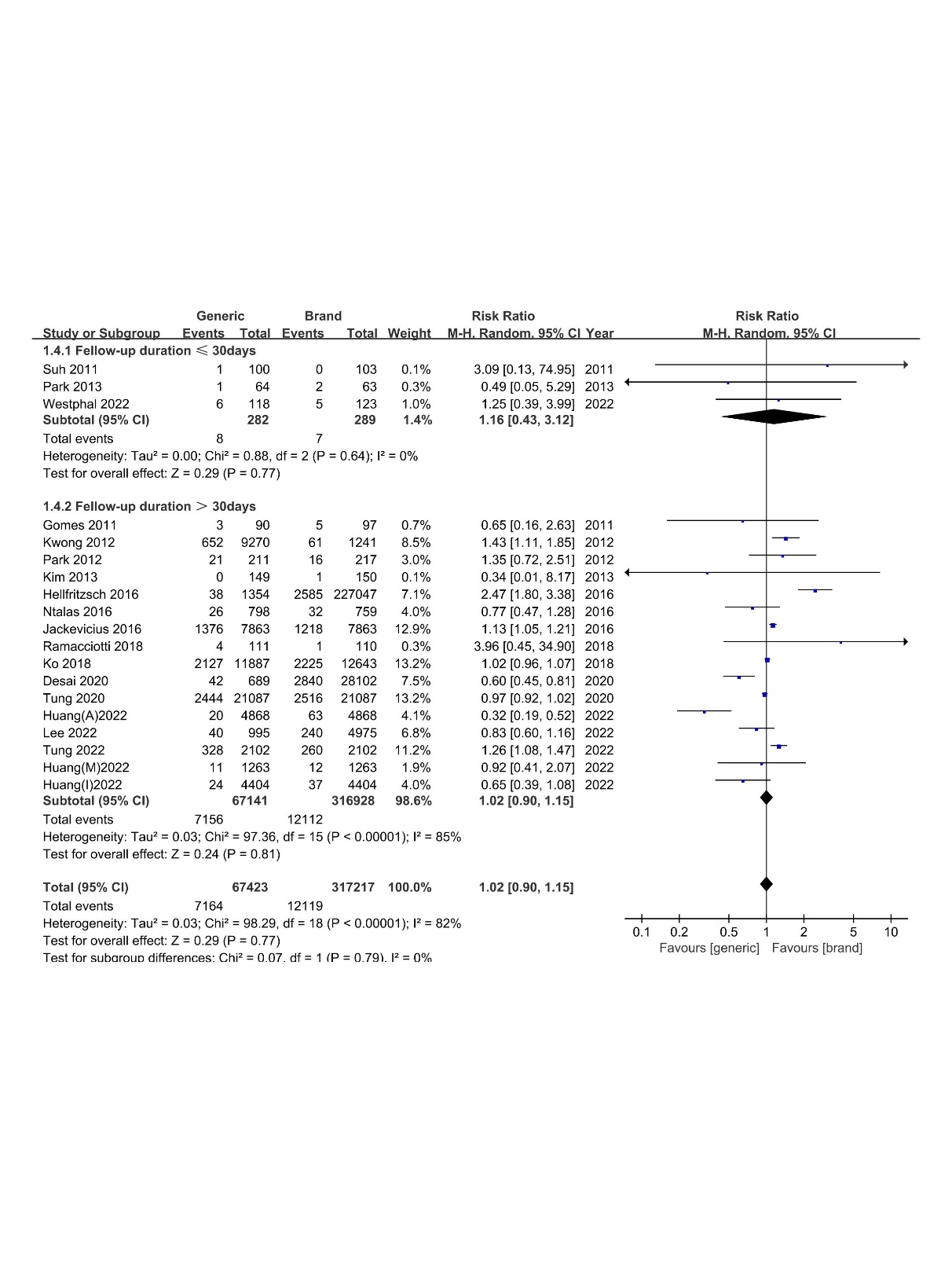


Supplementary Fig. 1 (D)Subgroup analysis according to funding source


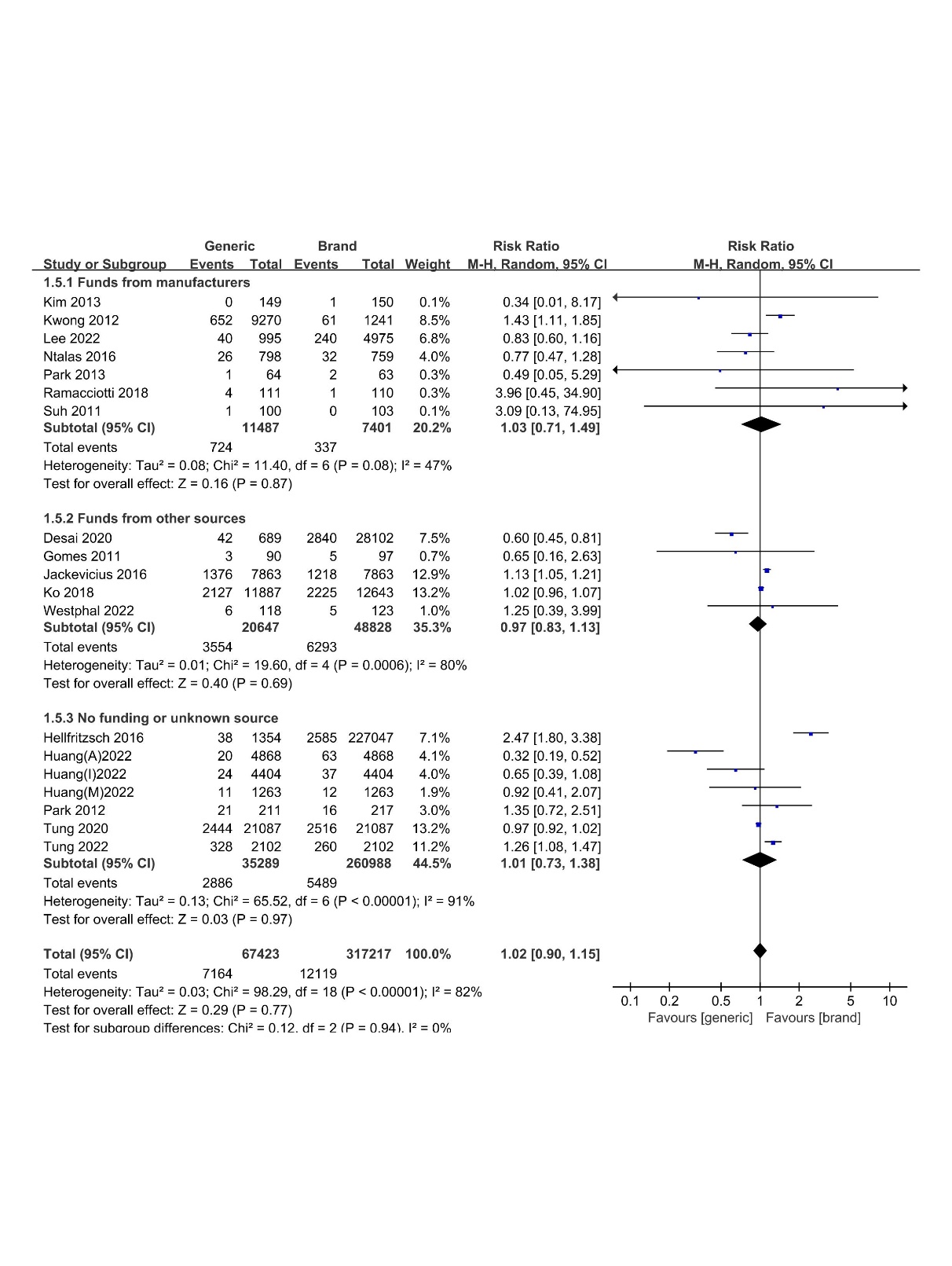

Supplement: Supplementary file 1 [file 2153-8174-26-3-26116-s1.zip › Supplementary Fig. 1.docx]
