# Supplementary material for: Can Generic Medications Be a Safe and Effective Alternative to Brand-Name Drugs for Cardiovascular Disease Treatment? A Systematic Review and Meta-Analysis
Source: Rev Cardiovasc Med. 2025 Mar 7;26(3):26116. doi: 10.31083/RCM26116 (PMC11951291; doi:10.31083/RCM26116)
Supplement: Supplementary file 1 [file 2153-8174-26-3-26116-s1.zip › Supplementary Fig. 3.docx]

Supplementary Fig. 3. Funnel plots

Supplementary Fig. 3 (a) Major adverse cardiovascular events


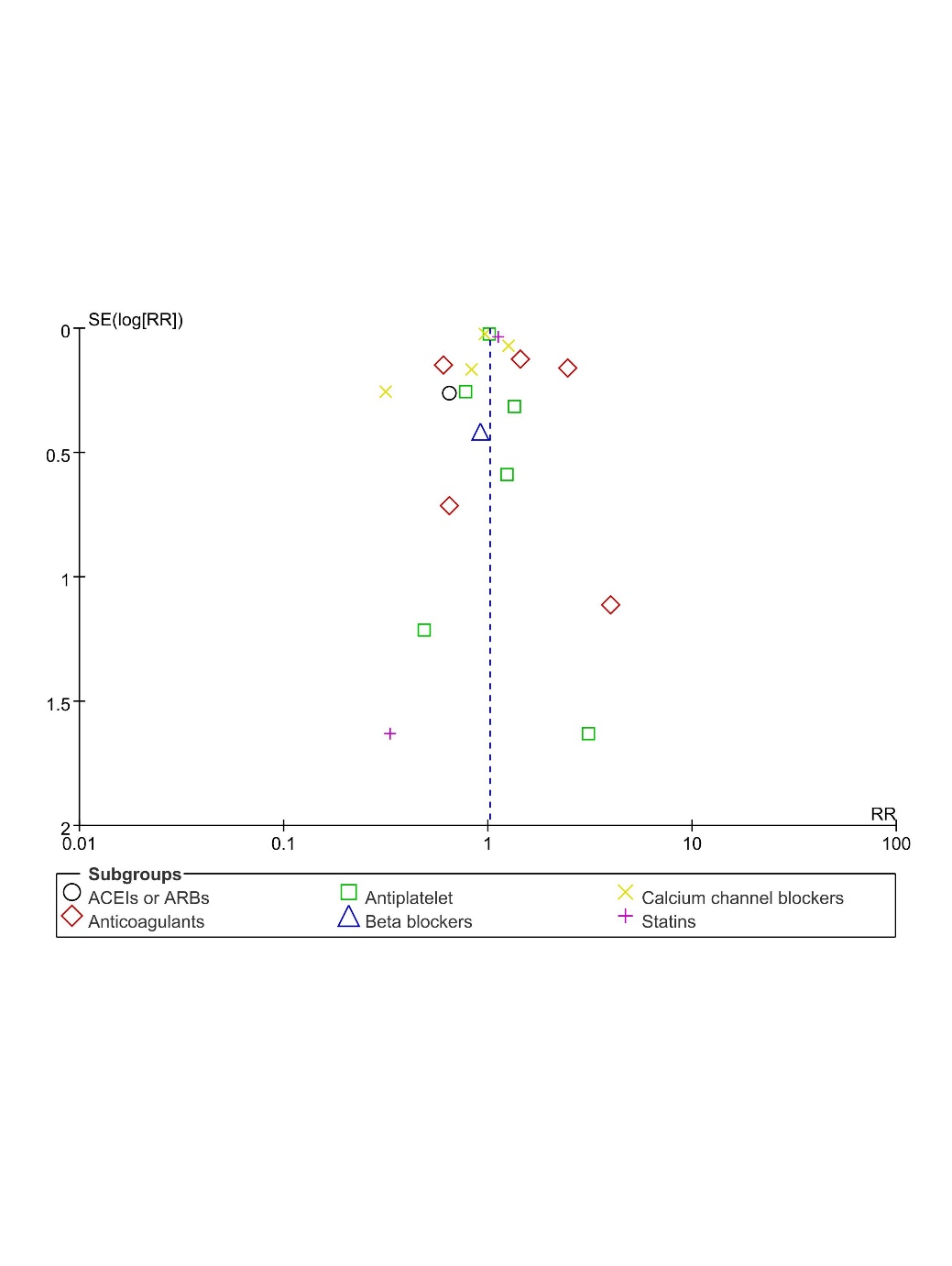


Supplementary Fig. 3. Funnel plots

Supplementary Fig. 3 (b) Adverse events

**
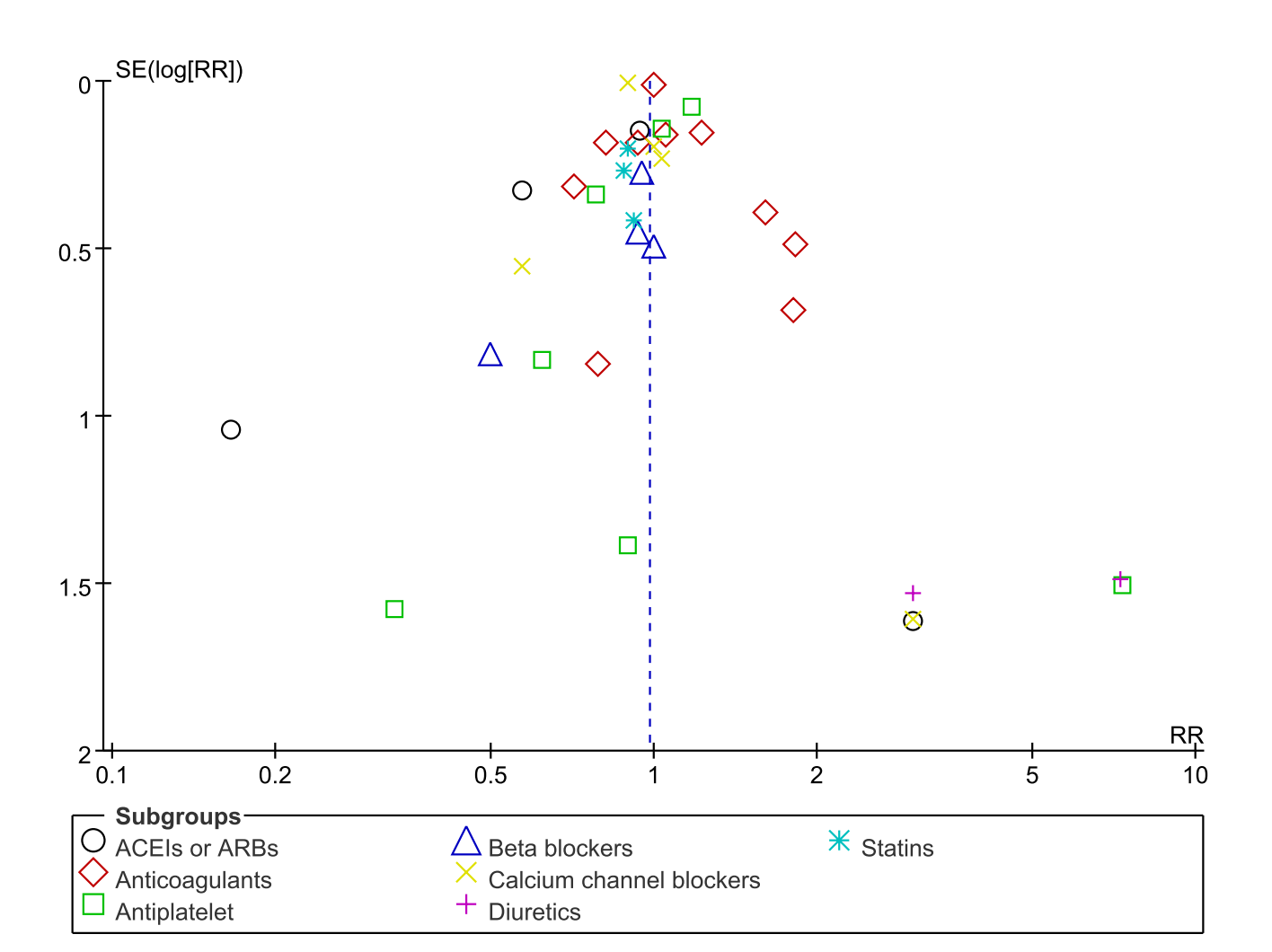
**
