# Supplementary material for: Can Generic Medications Be a Safe and Effective Alternative to Brand-Name Drugs for Cardiovascular Disease Treatment? A Systematic Review and Meta-Analysis
Source: Rev Cardiovasc Med. 2025 Mar 7;26(3):26116. doi: 10.31083/RCM26116 (PMC11951291; doi:10.31083/RCM26116)
Supplement: Supplementary file 1 [file 2153-8174-26-3-26116-s1.zip › Supplementary File 1. PRISMA 2020 flow diagram;Search strategy.docx]

**Additional File 1**

The Preferred Reporting Items for Systematic Reviews and Meta-Analyses (PRISMA) flow diagram

**Identification of studies via databases and registers**

Records removed *before screening*:

Duplicate records removed (n =312 )

Records marked as ineligible by automation tools (n =243 )

Records removed for other reasons

(n =23 )

Records identified from:

Databases (n =4230 )

Registers (n = 8)

**Identification**

Records excluded

(n = 3524)

Records screened

(n =3660 )

**Screening**

Reports excluded:

No mention of the original drug (n = 4)

Change of medication midway (n = 9)

Incomplete data (n = 32)

Studies included in quantitative synthesis (meta-analysis)

(n=54)

Reports assessed for eligibility

(n = 132)

Reports excluded:

Data cannot be extracted (n = 33)

Studies included in qualitative synthesis

(n=87)

**Included**

**Search Strategy**

**Items 1: Terms related to the study**

("Clinical study" or "Clinical trial" or "Cross-Over Studies" or "Cohort study" or " Randomized controlled trial" or "Parallel-group Studies")

"clinical study"[Publication Type] OR "clinical studies as topic"[MeSH Terms] OR "clinical study"[All Fields] OR ("clinical trial"[Publication Type] OR "clinical trials as topic"[MeSH Terms] OR "clinical trial"[All Fields]) OR ("cross over studies"[MeSH Terms] OR ("cross over"[All Fields] AND "studies"[All Fields]) OR "cross over studies"[All Fields] OR ("cross"[All Fields] AND "over"[All Fields] AND "studies"[All Fields]) OR "cross over studies"[All Fields]) OR ("cohort studies"[MeSH Terms] OR ("cohort"[All Fields] AND "studies"[All Fields]) OR "cohort studies"[All Fields] OR ("cohort"[All Fields] AND "study"[All Fields]) OR "cohort study"[All Fields]) OR ("randomized controlled trial"[Publication Type] OR "randomized controlled trials as topic"[MeSH Terms] OR "randomized controlled trial"[All Fields] OR "randomised controlled trial"[All Fields]) OR ("Parallel-group"[All Fields] AND ("studies"[All Fields] OR "study"[All Fields] OR "study s"[All Fields] OR "studying"[All Fields] OR "studys"[All Fields]))

**Items 2: Terms related to drugs**

("original drug " or "brand-name drug" or "innovator" or "patent drug" or "generic drug" or "non-brand drug" or "off-patent drug " or "other brands")

(("creativity"[MeSH Terms] OR "creativity"[All Fields] OR "originality"[All Fields] OR "original"[All Fields] OR "originalities"[All Fields] OR "originally"[All Fields] OR "originals"[All Fields] OR "originator"[All Fields] OR "originator s"[All Fields] OR "originators"[All Fields]) AND "drug"[All Fields]) OR ("brand-name"[All Fields] AND "drug"[All Fields]) OR ("creativity"[MeSH Terms] OR "creativity"[All Fields] OR "innovativeness"[All Fields] OR "innovate"[All Fields] OR "innovated"[All Fields] OR "innovates"[All Fields] OR "innovating"[All Fields] OR "innovation"[All Fields] OR "innovation s"[All Fields] OR "innovational"[All Fields] OR "innovations"[All Fields] OR "innovative"[All Fields] OR "innovatively"[All Fields] OR "innovator"[All Fields] OR "innovator s"[All Fields] OR "innovators"[All Fields]) OR (("patent"[All Fields] OR "patent s"[All Fields] OR "patentability"[All Fields] OR "patentable"[All Fields] OR "patented"[All Fields] OR "patenting"[All Fields] OR "patents"[All Fields]) AND "drug"[All Fields]) OR ("drugs, generic"[MeSH Terms] OR ("drugs"[All Fields] AND "generic"[All Fields]) OR "generic drugs"[All Fields] OR ("generic"[All Fields] AND "drug"[All Fields]) OR "generic drug"[All Fields]) OR ("non-brand"[All Fields] AND "drug"[All Fields]) OR ("off-patent"[All Fields] AND "drug"[All Fields]) OR ("other"[All Fields] AND ("brand"[All Fields] OR "brand s"[All Fields] OR "branded"[All Fields] OR "branding"[All Fields] OR "brands"[All Fields]))

**Items 3: Terms related to cardiovascular disease**

("Cardiovascular disease" or "coronary heart disease" or "ischemic heart disease" or "acute coronary syndrome" or "myocardial infarction" or "angina pectoris" or "atrial fibrillation" or "atrial flutter" or "heart failure" or "congestive heart disease" or "hypertension" or "hypercholesterolemia" or "atherosclerosis")

"cardiovascular diseases"[MeSH Terms] OR ("cardiovascular"[All Fields] AND "diseases"[All Fields]) OR "cardiovascular diseases"[All Fields] OR ("cardiovascular"[All Fields] AND "disease"[All Fields]) OR "cardiovascular disease"[All Fields] OR ("coronary disease"[MeSH Terms] OR ("coronary"[All Fields] AND "disease"[All Fields]) OR "coronary disease"[All Fields] OR ("coronary"[All Fields] AND "heart"[All Fields] AND "disease"[All Fields]) OR "coronary heart disease"[All Fields]) OR ("ischaemic heart disease"[All Fields] OR "myocardial ischemia"[MeSH Terms] OR ("myocardial"[All Fields] AND "ischemia"[All Fields]) OR "myocardial ischemia"[All Fields] OR ("ischemic"[All Fields] AND "heart"[All Fields] AND "disease"[All Fields]) OR "ischemic heart disease"[All Fields] OR "coronary artery disease"[MeSH Terms] OR ("coronary"[All Fields] AND "artery"[All Fields] AND "disease"[All Fields]) OR "coronary artery disease"[All Fields]) OR ("acute coronary syndrome"[MeSH Terms] OR ("acute"[All Fields] AND "coronary"[All Fields] AND "syndrome"[All Fields]) OR "acute coronary syndrome"[All Fields]) OR ("myocardial infarction"[MeSH Terms] OR ("myocardial"[All Fields] AND "infarction"[All Fields]) OR "myocardial infarction"[All Fields]) OR ("angina pectoris"[MeSH Terms] OR ("angina"[All Fields] AND "pectoris"[All Fields]) OR "angina pectoris"[All Fields]) OR ("atrial fibrillation"[MeSH Terms] OR ("atrial"[All Fields] AND "fibrillation"[All Fields]) OR "atrial fibrillation"[All Fields]) OR ("atrial flutter"[MeSH Terms] OR ("atrial"[All Fields] AND "flutter"[All Fields]) OR "atrial flutter"[All Fields]) OR ("heart failure"[MeSH Terms] OR ("heart"[All Fields] AND "failure"[All Fields]) OR "heart failure"[All Fields]) OR ("heart failure"[MeSH Terms] OR ("heart"[All Fields] AND "failure"[All Fields]) OR "heart failure"[All Fields] OR ("congestive"[All Fields] AND "heart"[All Fields] AND "disease"[All Fields]) OR "congestive heart disease"[All Fields]) OR ("hypertense"[All Fields] OR "hypertension"[MeSH Terms] OR "hypertension"[All Fields] OR "hypertension s"[All Fields] OR "hypertensions"[All Fields] OR "hypertensive"[All Fields] OR "hypertensive s"[All Fields] OR "hypertensives"[All Fields]) OR ("hypercholesterolaemia"[All Fields] OR "hypercholesterolemia"[MeSH Terms] OR "hypercholesterolemia"[All Fields] OR "hypercholesterolaemias"[All Fields] OR "hypercholesterolemias"[All Fields]) OR ("atherosclerosis"[MeSH Terms] OR "atherosclerosis"[All Fields] OR "atheroscleroses"[All Fields])

**Items 4: Terms related to medication**

("angiotensin converting enzyme inhibitors" or "angiotensin receptor blockers" or "antihypertensive drugs" or "beta-blockers" or "calcium channel blockers" or "antithrombotic drugs" or "antiplatelet drugs" or "anticoagulants" or "diuretics" or "statins")

"angiotensin converting enzyme inhibitors"[Pharmacological Action] OR "angiotensin converting enzyme inhibitors"[MeSH Terms] OR ("angiotensin converting"[All Fields] AND "enzyme"[All Fields] AND "inhibitors"[All Fields]) OR "angiotensin converting enzyme inhibitors"[All Fields] OR ("angiotensin"[All Fields] AND "converting"[All Fields] AND "enzyme"[All Fields] AND "inhibitors"[All Fields]) OR "angiotensin converting enzyme inhibitors"[All Fields] OR ("angiotensin receptor antagonists"[Pharmacological Action] OR "angiotensin receptor antagonists"[MeSH Terms] OR ("angiotensin"[All Fields] AND "receptor"[All Fields] AND "antagonists"[All Fields]) OR "angiotensin receptor antagonists"[All Fields] OR ("angiotensin"[All Fields] AND "receptor"[All Fields] AND "blockers"[All Fields]) OR "angiotensin receptor blockers"[All Fields]) OR ("antihypertensive agents"[Pharmacological Action] OR "antihypertensive agents"[MeSH Terms] OR ("antihypertensive"[All Fields] AND "agents"[All Fields]) OR "antihypertensive agents"[All Fields] OR ("antihypertensive"[All Fields] AND "drugs"[All Fields]) OR "antihypertensive drugs"[All Fields]) OR ("adrenergic beta antagonists"[Pharmacological Action] OR "adrenergic beta antagonists"[MeSH Terms] OR ("adrenergic"[All Fields] AND "beta antagonists"[All Fields]) OR "adrenergic beta antagonists"[All Fields] OR ("beta"[All Fields] AND "blockers"[All Fields]) OR "beta blockers"[All Fields]) OR ("calcium channel blockers"[Pharmacological Action] OR "calcium channel blockers"[MeSH Terms] OR ("calcium"[All Fields] AND "channel"[All Fields] AND "blockers"[All Fields]) OR "calcium channel blockers"[All Fields]) OR (("antithrombotic"[All Fields] OR "antithrombotics"[All Fields]) AND ("drug s"[All Fields] OR "pharmaceutical preparations"[MeSH Terms] OR ("pharmaceutical"[All Fields] AND "preparations"[All Fields]) OR "pharmaceutical preparations"[All Fields] OR "drugs"[All Fields])) OR ("platelet aggregation inhibitors"[Pharmacological Action] OR "platelet aggregation inhibitors"[MeSH Terms] OR ("platelet"[All Fields] AND "aggregation"[All Fields] AND "inhibitors"[All Fields]) OR "platelet aggregation inhibitors"[All Fields] OR ("antiplatelet"[All Fields] AND "drugs"[All Fields]) OR "antiplatelet drugs"[All Fields]) OR ("anticoagulants"[Pharmacological Action] OR "anticoagulants"[MeSH Terms] OR "anticoagulants"[All Fields] OR "anticoagulant"[All Fields] OR "anticoagulate"[All Fields] OR "anticoagulated"[All Fields] OR "anticoagulating"[All Fields] OR "anticoagulation"[All Fields] OR "anticoagulations"[All Fields] OR "anticoagulative"[All Fields]) OR ("diuretics"[Pharmacological Action] OR "diuretics"[MeSH Terms] OR "diuretics"[All Fields] OR "diuretic"[All Fields]) OR ("hydroxymethylglutaryl coa reductase inhibitors"[Pharmacological Action] OR "hydroxymethylglutaryl coa reductase inhibitors"[MeSH Terms] OR ("hydroxymethylglutaryl coa"[All Fields] AND "reductase"[All Fields] AND "inhibitors"[All Fields]) OR "hydroxymethylglutaryl coa reductase inhibitors"[All Fields] OR "statin"[All Fields] OR "statins"[All Fields] OR "statin s"[All Fields] OR "statine"[Supplementary Concept] OR "statine"[All Fields] OR "statines"[All Fields])
