# Supplementary material for: Can Generic Medications Be a Safe and Effective Alternative to Brand-Name Drugs for Cardiovascular Disease Treatment? A Systematic Review and Meta-Analysis
Source: Rev Cardiovasc Med. 2025 Mar 7;26(3):26116. doi: 10.31083/RCM26116 (PMC11951291; doi:10.31083/RCM26116)
Supplement: Supplementary file 1 [file 2153-8174-26-3-26116-s1.zip › Supplementary File 2 Subgroup analysis; Risk assessment of bias.docx]

**Additional File 2**

**Figure S1**. Subgroup analysis comparing major adverse cardiovascular events between the two groups

**Figure S2**. Subgroup analysis comparing adverse events between the two groups

**Table S1**. Risk assessment of bias in included studies

**Figure S3**. Funnel plots

Figure S1. Subgroup analysis comparing major adverse cardiovascular events (MACE) between the two groups

Figure S1 (A) Subgroup analysis according to region


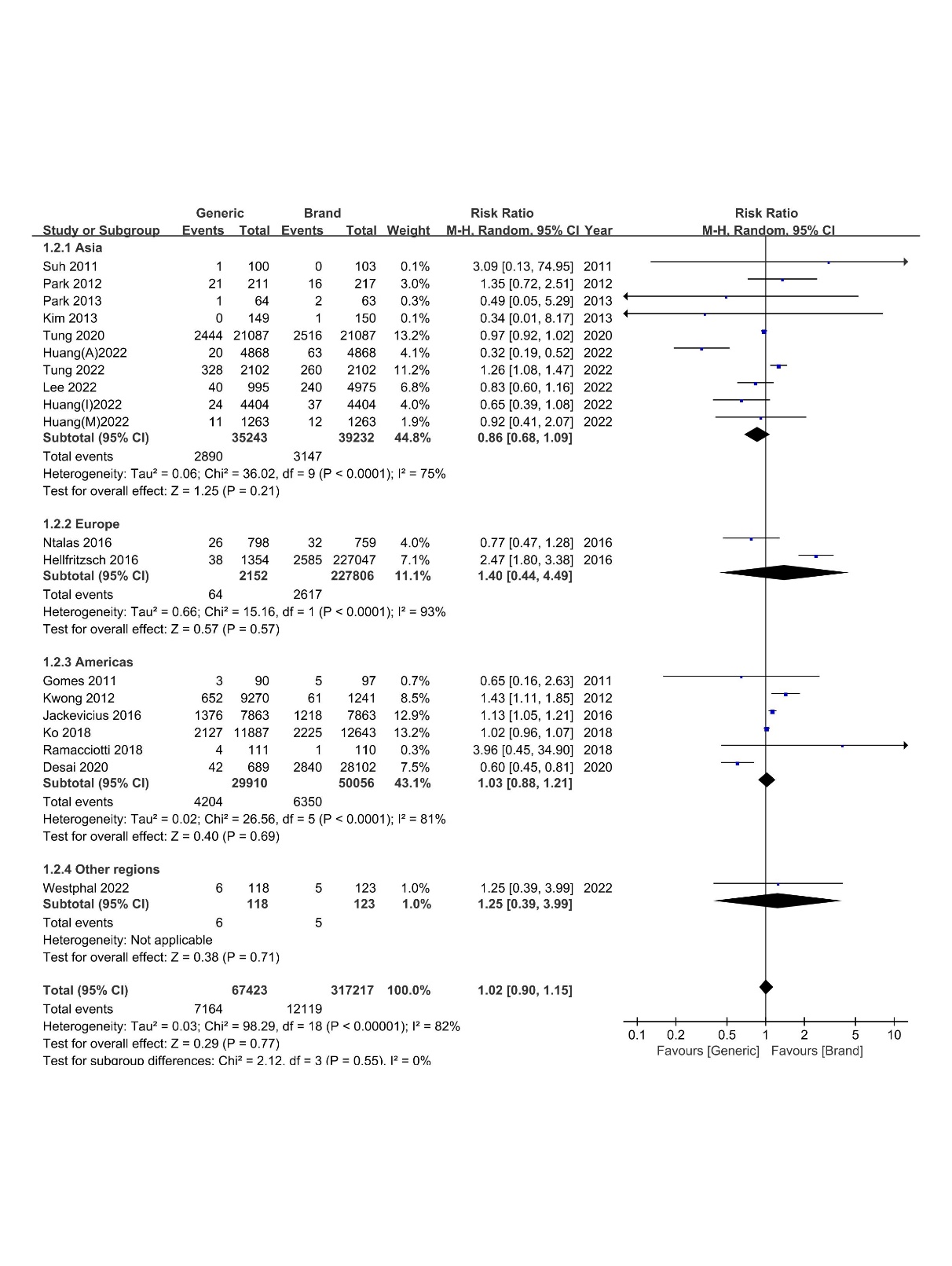


Figure S1 (B) Subgroup analysis according to study design


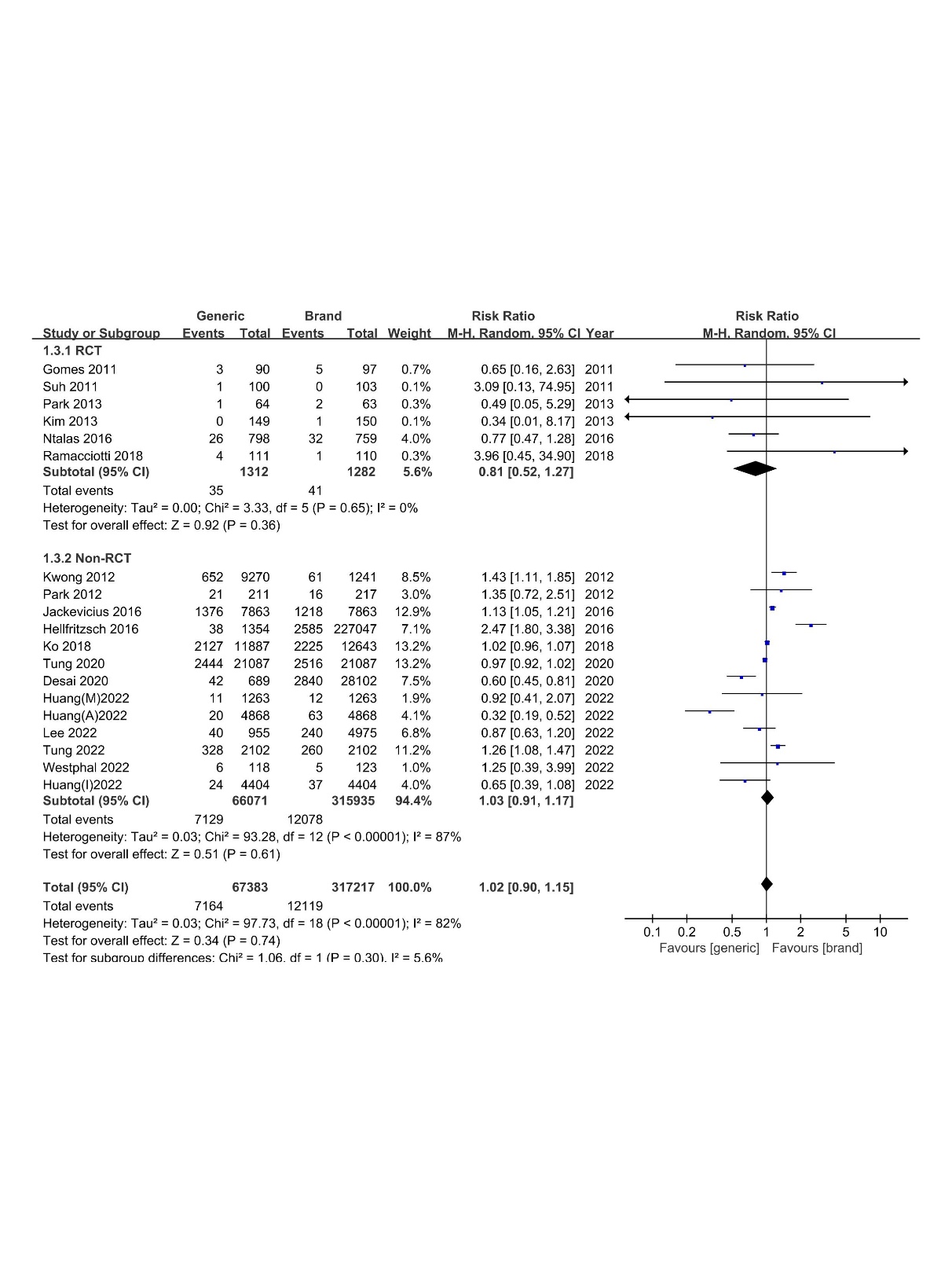


Figure S1 (C) Subgroup analysis according to the follow-up time


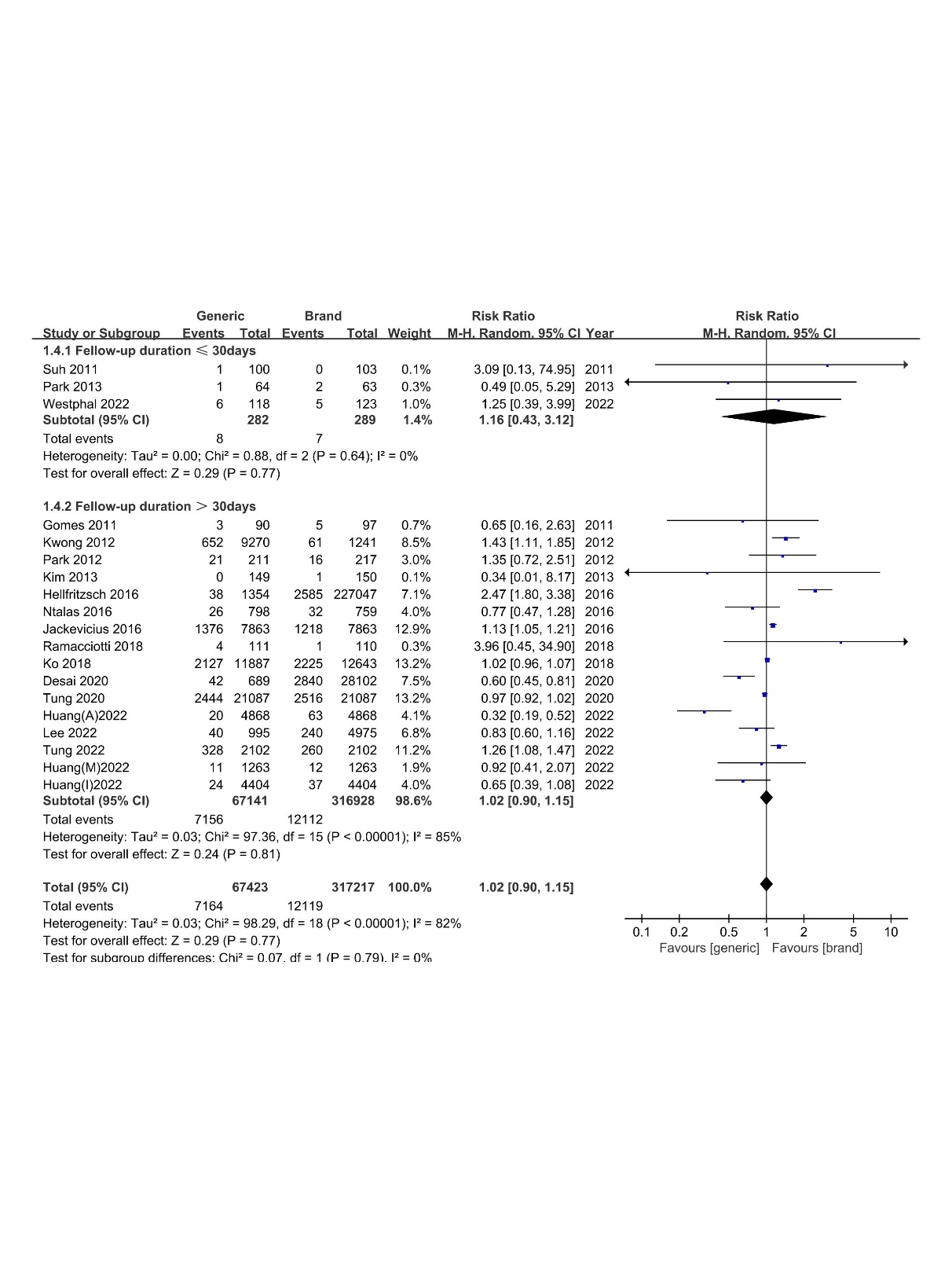


Figure S1 (D)Subgroup analysis according to funding source


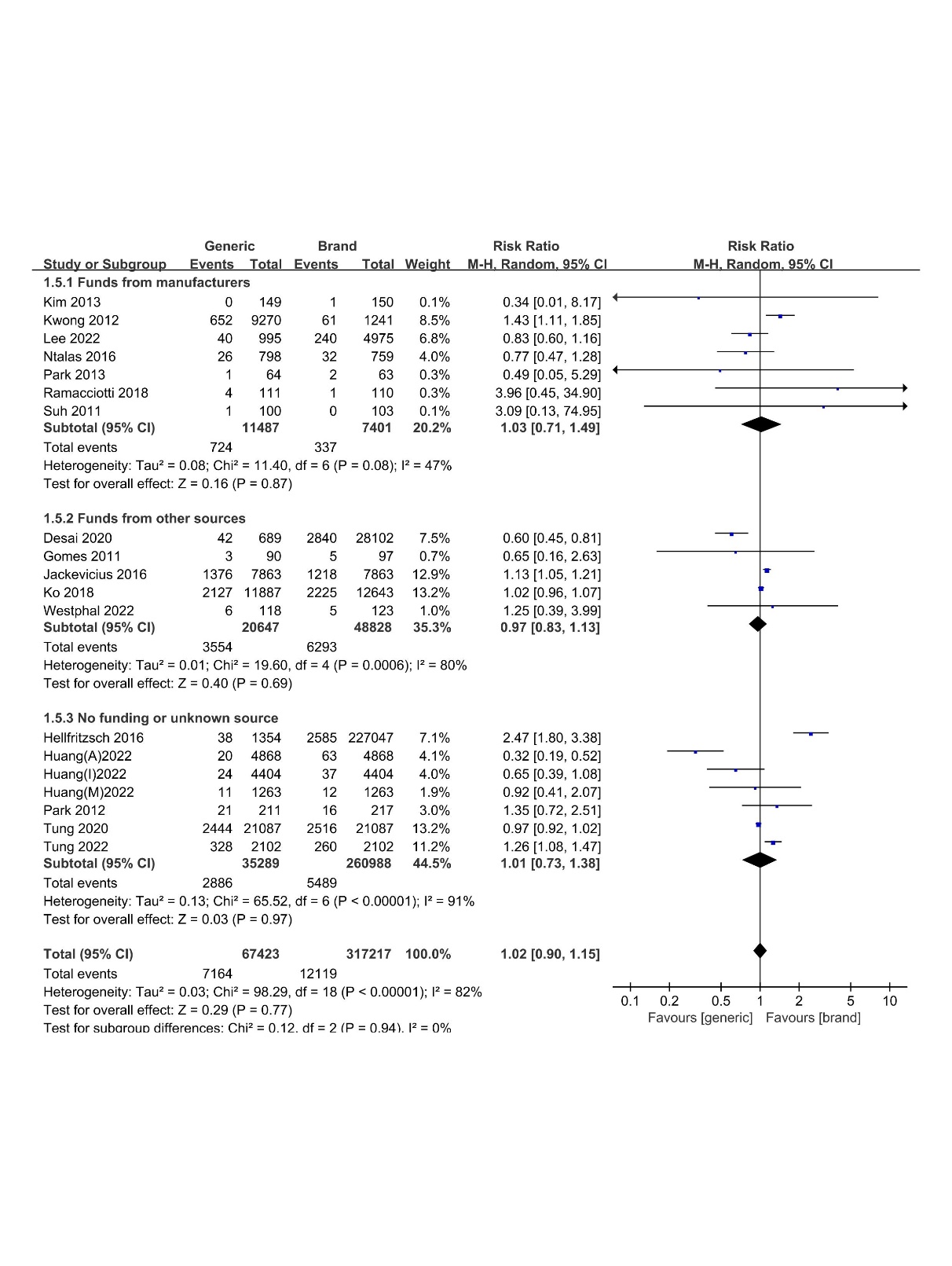


Figure S2. Subgroup analysis comparing adverse events (AEs) between the two groups

Figure S2 (A) Subgroup analysis according to region


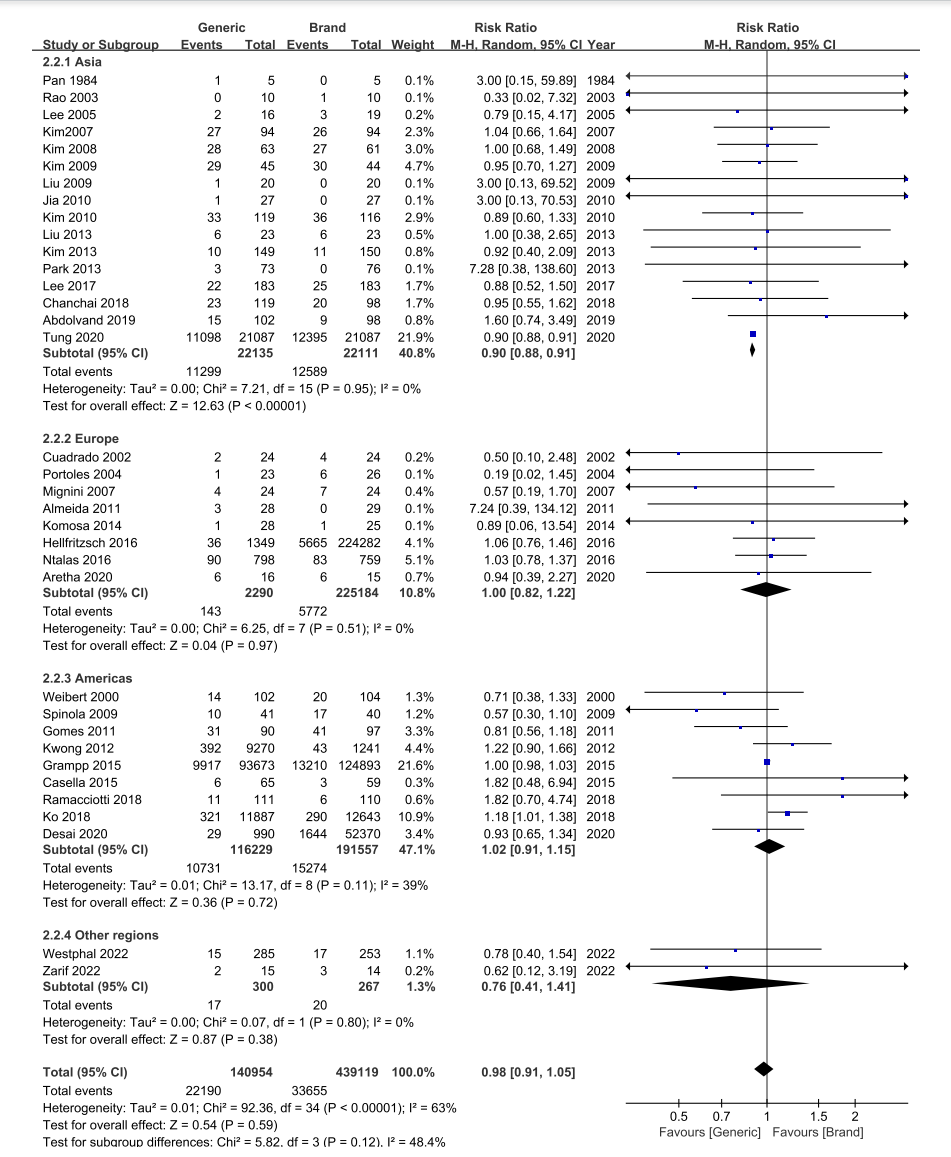


Figure S2 (B) Subgroup analysis according to study design


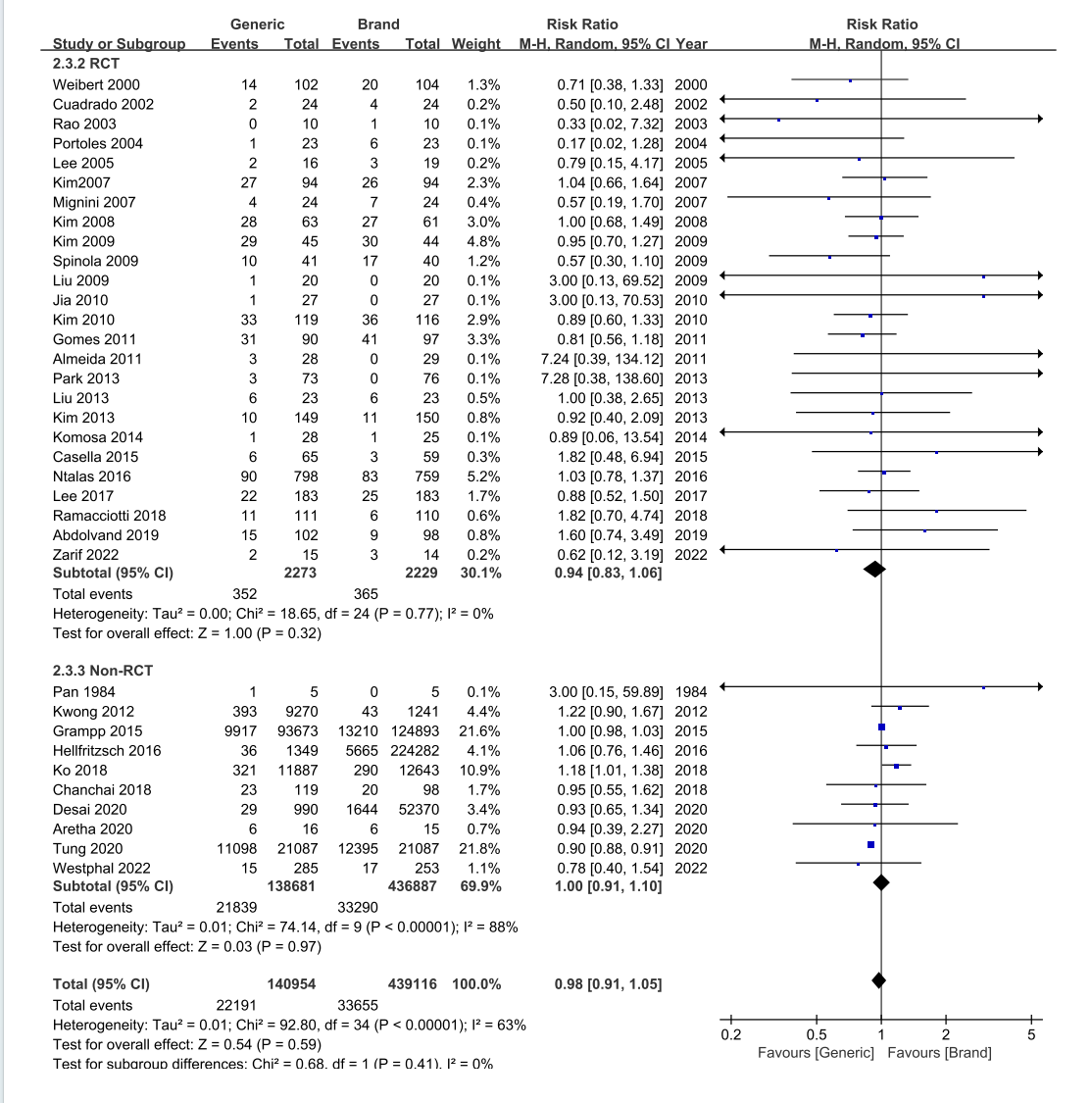


Figure S2 (C) Subgroup analysis according to the follow-up time


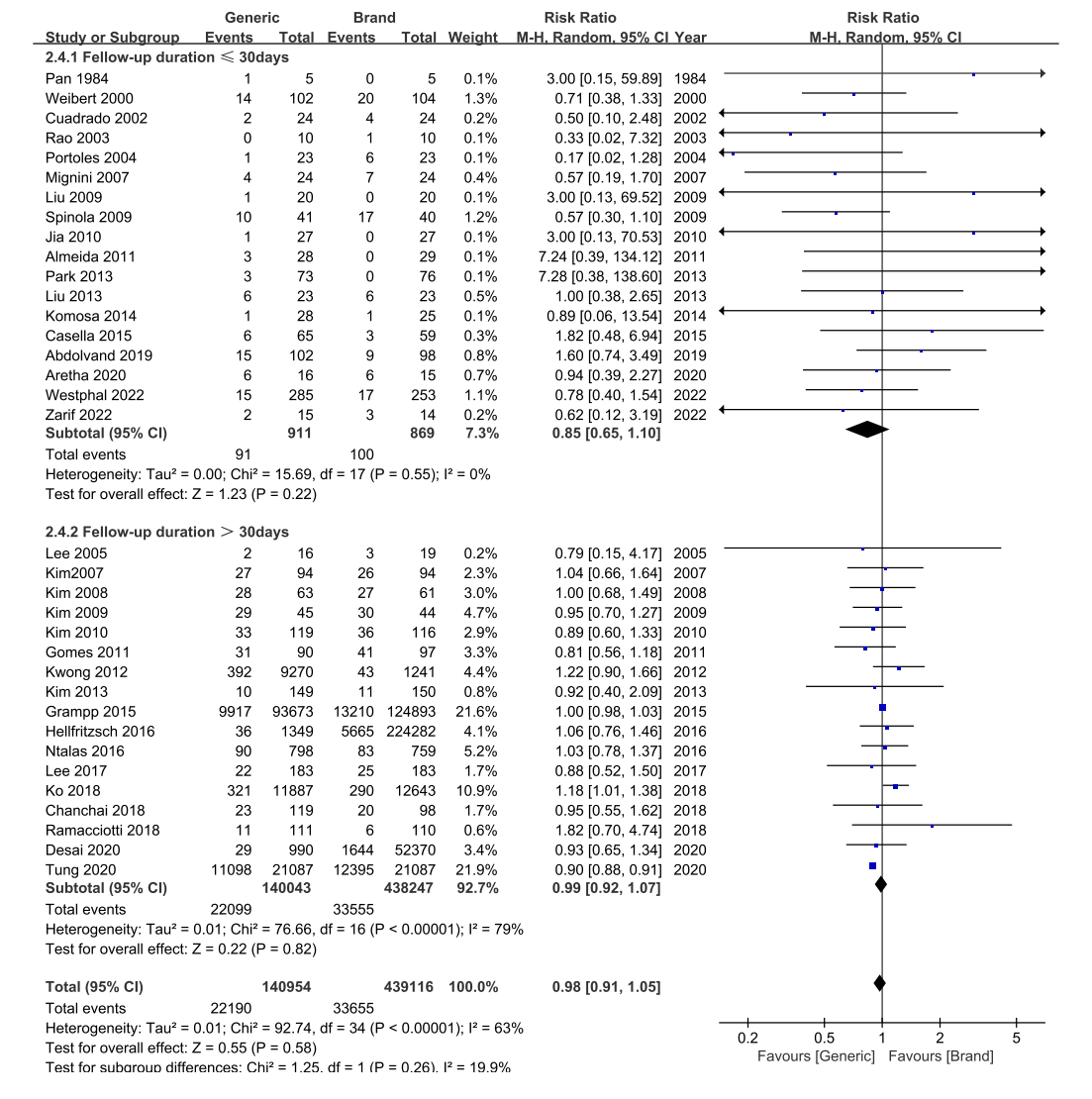


Figure S2 (D)Subgroup analysis according to funding source


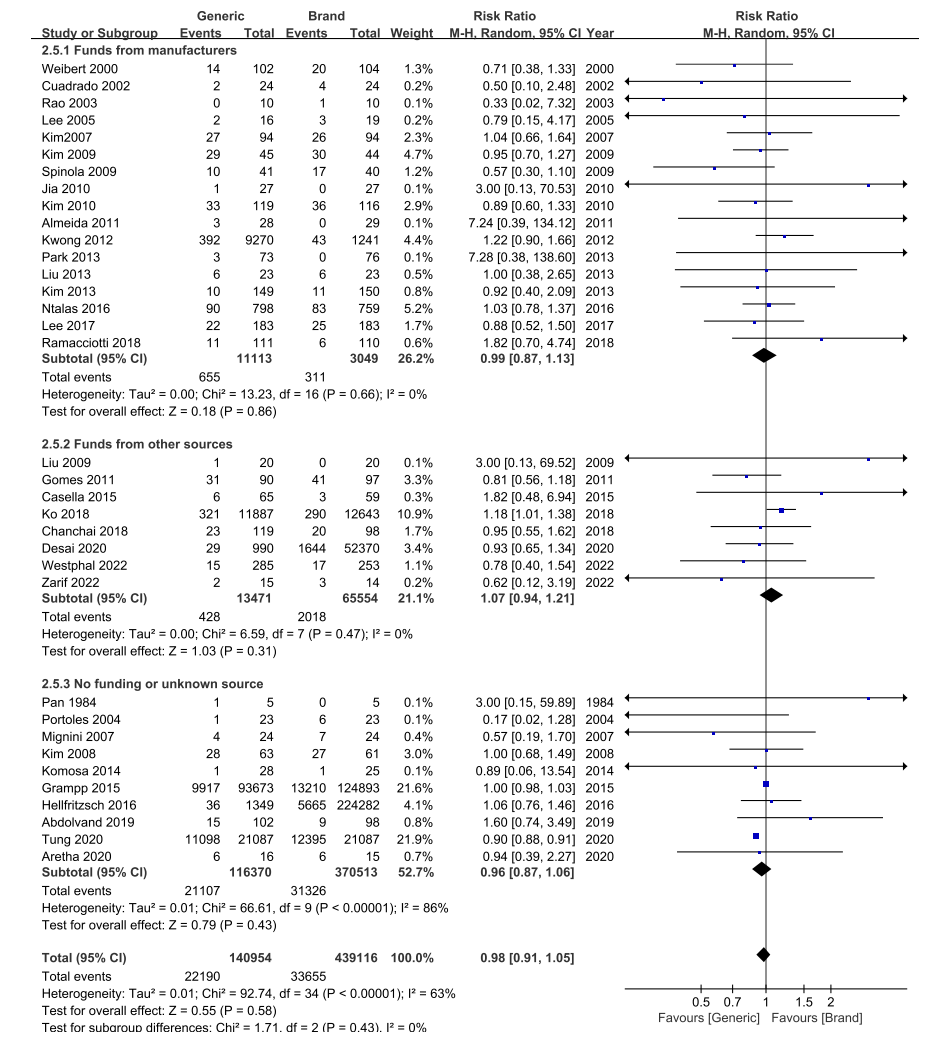


Table S1. Risk assessment of bias in included studies

Table S1（a）Risk assessment of bias in RCTs

| Author / Year  （Classification by drug） | Random sequence generation | Allocation sequence  concealment | Blind assessment | Incomplete outcome data | Selective reporting of results | Other sources of bias |
| --- | --- | --- | --- | --- | --- | --- |
| **ACE inhibitors or ARBs** |  |  |  |  |  |  |
| Portoles 2004 | Y | N | N | Y | Y | Y |
| Kim 2009 | UC | N | N | Y | N | N |
| Spinola 2009 | Y | Y | N | Y | Y | Y |
| Iqbal 2010 | Y | N | N | N | N | N |
| Jia 2010 | Y | N | N | Y | N | N |
| Li 2010 | Y | N | N | Y | Y | Y |
| Oigman 2013 | UC | N | N | Y | N | N |
| Patel 2017 | UC | N | N | Y | Y | Y |
| **Anticoagulants** |  |  |  |  |  |  |
| Weibert 2000 | Y | N | N | N | N | N |
| Lee 2005 | Y | Y | N | Y | N | N |
| Pereira 2005 | UC | Y | Y | Y | N | N |
| Gomes 2011 | UC | N | N | Y | Y | N |
| Ramacciotti 2018 | Y | Y | Y | Y | Y | N |
| Abdolvand 2019 | UC | Y | N | Y | Y | N |
| Casella 2015 | UC | UC | N | Y | Y | N |
| Gomes 2021 | UC | UC | Y | Y | Y | N |
| **Antiplatelet agents** |  |  |  |  |  |  |
| Rao 2003 | Y | N | N | Y | Y | Y |
| Kim (P) 2009 | Y | N | N | Y | N | N |
| Di 2010 | Y | N | N | Y | N | N |
| Müller 2010 | UC | UC | UC | Y | Y | Y |
| Shim 2010 | Y | N | N | Y | N | N |
| Khosravi 2011 | Y | N | Y | Y | Y | Y |
| Suh 2011 | Y | N | Y | Y | Y | Y |
| Oberhänsli 2012 | Y | N | Y | Y | Y | Y |
| Tsoumani (A) 2012 | UC | N | N | Y | N | N |
| Tsoumani (E) 2012 | UC | N | N | Y | N | N |
| Park 2013 | Y | Y | N | Y | Y | N |
| Komosa 2014 | Y | N | N | Y | N | Y |
| Seo 2014 | UC | N | N | Y | Y | N |
| Hamilos 2015 | Y | Y | N | Y | Y | N |
| Ntalas 2016 | Y | UC | N | Y | N | N |
| Hajizadeh 2017 | UC | UC | Y | Y | Y | Y |
| Zarif 2022 | Y | UC | UC | Y | Y | Y |
| **Beta-blockers** |  |  |  |  |  |  |
| Carter 1989 | Y | N | N | Y | Y | N |
| el-Sayed 1989 | Y | N | Y | Y | N | N |
| Sarkar 1995 | UC | N | Y | Y | N | N |
| Cuadrado 2002 | UC | N | N | Y | N | N |
| Portoles 2005 | UC | UC | N | Y | N | N |
| Liu 2013 | Y | UC | N | Y | N | Y |
| Mosley 2022 | Y | N | Y | Y | Y | N |
| **Calcium channel blockers** |  |  |  |  |  |  |
| Saseen 1997 | UC | UC | Y | Y | N | Y |
| Usha 1997 | UC | N | Y | Y | N | N |
| Park 2004 | Y | UC | N | Y | Y | Y |
| Kim 2007 | Y | Y | Y | Y | Y | Y |
| Mignini 2007 | Y | UC | N | Y | N | Y |
| Kim 2008 | Y | Y | Y | Y | Y | Y |
| Liu 2009 | Y | UC | N | Y | N | N |
| Pollak 2017 | Y | Y | N | Y | Y | Y |
| **Diuretics** |  |  |  |  |  |  |
| Murray 1997 | UC | N | N | Y | Y | Y |
| Almeida 2011 | Y | Y | UC | Y | N | Y |
| **Statins** |  |  |  |  |  |  |
| Wiwanitkit 2002 | UC | Y | Y | Y | Y | Y |
| Liu 2010 | Y | N | N | Y | Y | N |
| Kim 2010 | Y | Y | Y | Y | Y | Y |
| Kim 2013 | Y | UC | N | Y | Y | Y |
| Lee 2017 | Y | UC | N | Y | Y | N |
| Kim 2020 | UC | UC | N | Y | Y | N |

Y: low risk of bias; N: high risk of bias; UC: uncertainty about bias or lack of necessary information.

Table S1. Risk assessment of bias in included studies

Table S1 (b) Risk assessment of bias in non-randomized clinical studies

| Author / Year  （Classification by drug） | Confounding bias | selection bias | Bias in measurement classification of interventions | Bias due to deviations from intended interventions | Bias due to missing data | Bias in measurement of outcomes | Bias in selection of the reported result |
| --- | --- | --- | --- | --- | --- | --- | --- |
| **ACE inhibitors or ARBs** |  |  |  |  |  |  |  |
| Leclerc 2017 | M | M | L | L | M | S | L |
| Huang（I）2022 | L | M | L | L | M | M | L |
| **Anticoagulants** |  |  |  |  |  |  |  |
| Kwong 2012 | M | L | M | M | L | M | L |
| Hellfritzsch 2016 | L | L | M | L | L | L | L |
| Leclerc 2018 | L | L | M | L | M | M | L |
| Grampp 2015 | L | L | L | L | M | M | M |
| Desai 2020 | M | L | L | L | M | M | L |
| Fantoni 2021 | L | M | L | L | M | L | L |
| Feng 2009 | L | M | L | L | L | L | L |
| **Antiplatelet agents** |  |  |  |  |  |  |  |
| Park 2012 | M | L | L | M | M | M | L |
| Kovacic 2014 | L | L | L | L | L | M | M |
| Westphal 2022 | L | L | L | L | L | L | L |
| Ko 2018 | M | S | L | L | M | M | L |
| Leclerc 2019 | M | M | S | L | S | L | M |
| Patsourakos2020 | L | M | L | L | L | L | L |
| **Beta-blockers** |  |  |  |  |  |  |  |
| Ahrens 2007 | M | L | S | S | M | M | M |
| Chanchai 2018 | M | M | L | L | L | L | L |
| Huang(M) 2022 | M | M | L | L | M | L | L |
| Aretha 2020 | L | L | L | M | L | L | L |
| **Calcium channel blockers** |  |  |  |  |  |  |  |
| Desai2019 | M | M | L | L | M | L | L |
| Huang（A）2022 | L | L | L | L | M | M | M |
| Tung 2020 | NI | M | L | L | NI | NI | L |
| Lee 2022 | M | S | M | M | L | NI | L |
| Tung2022 | M | NI | L | L | M | NI | L |
| **Diuretics** |  |  |  |  |  |  |  |
| Martin 1984 | L | L | L | L | NI | L | L |
| Pan 1984 | L | L | L | L | NI | L | L |
| **Statins** |  |  |  |  |  |  |  |
| Corrao 2014 | M | L | NI | M | M | L | L |
| Gagne 2014 | L | L | M | M | M | M | L |
| Jackevicius 2016 | L | L | L | NI | NI | L | L |
| Sicras-Mainar2018 | NI | M | M | NI | M | L | L |
| Manasirisuk 2021 | L | L | NI | NI | L | M | L |

L: Low risk; M: Moderate risk; S: Serious risk; C: Critical risk; NI: No information.

Figure S3. Funnel plots

Figure S3 (a) Major adverse cardiovascular events


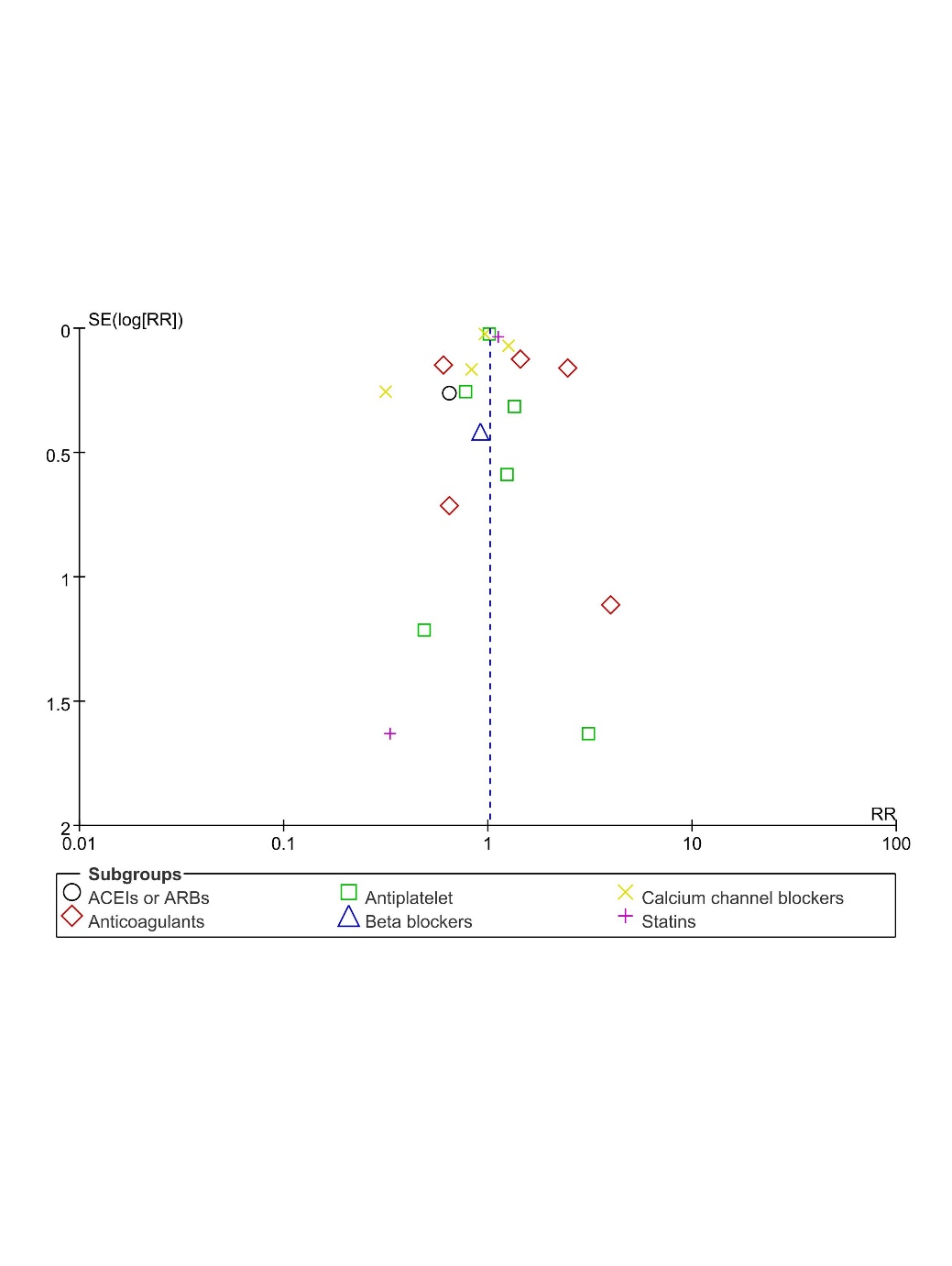


Fig S3. Funnel plots

Fig S3 (b) Adverse events

**
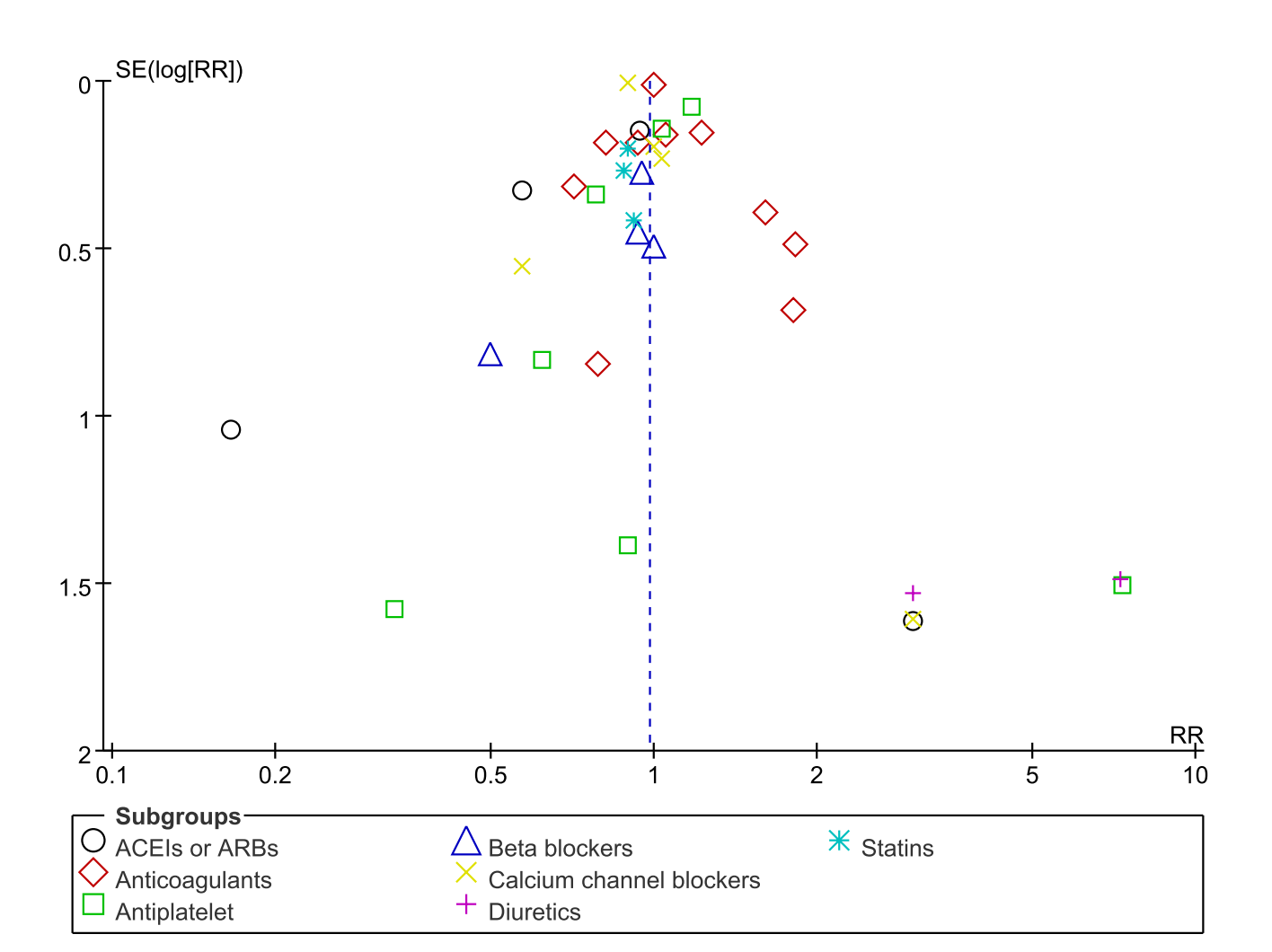
**
