# Supplementary material for: Can Generic Medications Be a Safe and Effective Alternative to Brand-Name Drugs for Cardiovascular Disease Treatment? A Systematic Review and Meta-Analysis
Source: Rev Cardiovasc Med. 2025 Mar 7;26(3):26116. doi: 10.31083/RCM26116 (PMC11951291; doi:10.31083/RCM26116)
Supplement: Supplementary file 1 [file 2153-8174-26-3-26116-s1.zip › Supplementary Table 1.docx]

Supplementary Table 1. Risk assessment of bias in included studies

Supplementary Table 1（a）Risk assessment of bias in RCTs

| Author / Year  （Classification by drug） | Random sequence generation | Allocation sequence  concealment | Blind assessment | Incomplete outcome data | Selective reporting of results | Other sources of bias |
| --- | --- | --- | --- | --- | --- | --- |
| **ACE inhibitors or ARBs** |  |  |  |  |  |  |
| Portoles 2004 | Y | N | N | Y | Y | Y |
| Kim 2009 | UC | N | N | Y | N | N |
| Spinola 2009 | Y | Y | N | Y | Y | Y |
| Iqbal 2010 | Y | N | N | N | N | N |
| Jia 2010 | Y | N | N | Y | N | N |
| Li 2010 | Y | N | N | Y | Y | Y |
| Oigman 2013 | UC | N | N | Y | N | N |
| Patel 2017 | UC | N | N | Y | Y | Y |
| **Anticoagulants** |  |  |  |  |  |  |
| Weibert 2000 | Y | N | N | N | N | N |
| Lee 2005 | Y | Y | N | Y | N | N |
| Pereira 2005 | UC | Y | Y | Y | N | N |
| Gomes 2011 | UC | N | N | Y | Y | N |
| Ramacciotti 2018 | Y | Y | Y | Y | Y | N |
| Abdolvand 2019 | UC | Y | N | Y | Y | N |
| Casella 2015 | UC | UC | N | Y | Y | N |
| Gomes 2021 | UC | UC | Y | Y | Y | N |
| **Antiplatelet agents** |  |  |  |  |  |  |
| Rao 2003 | Y | N | N | Y | Y | Y |
| Kim (P) 2009 | Y | N | N | Y | N | N |
| Di 2010 | Y | N | N | Y | N | N |
| Müller 2010 | UC | UC | UC | Y | Y | Y |
| Shim 2010 | Y | N | N | Y | N | N |
| Khosravi 2011 | Y | N | Y | Y | Y | Y |
| Suh 2011 | Y | N | Y | Y | Y | Y |
| Oberhänsli 2012 | Y | N | Y | Y | Y | Y |
| Tsoumani (A) 2012 | UC | N | N | Y | N | N |
| Tsoumani (E) 2012 | UC | N | N | Y | N | N |
| Park 2013 | Y | Y | N | Y | Y | N |
| Komosa 2014 | Y | N | N | Y | N | Y |
| Seo 2014 | UC | N | N | Y | Y | N |
| Hamilos 2015 | Y | Y | N | Y | Y | N |
| Ntalas 2016 | Y | UC | N | Y | N | N |
| Hajizadeh 2017 | UC | UC | Y | Y | Y | Y |
| Zarif 2022 | Y | UC | UC | Y | Y | Y |
| **Beta-blockers** |  |  |  |  |  |  |
| Carter 1989 | Y | N | N | Y | Y | N |
| el-Sayed 1989 | Y | N | Y | Y | N | N |
| Sarkar 1995 | UC | N | Y | Y | N | N |
| Cuadrado 2002 | UC | N | N | Y | N | N |
| Portoles 2005 | UC | UC | N | Y | N | N |
| Liu 2013 | Y | UC | N | Y | N | Y |
| Mosley 2022 | Y | N | Y | Y | Y | N |
| **Calcium channel blockers** |  |  |  |  |  |  |
| Saseen 1997 | UC | UC | Y | Y | N | Y |
| Usha 1997 | UC | N | Y | Y | N | N |
| Park 2004 | Y | UC | N | Y | Y | Y |
| Kim 2007 | Y | Y | Y | Y | Y | Y |
| Mignini 2007 | Y | UC | N | Y | N | Y |
| Kim 2008 | Y | Y | Y | Y | Y | Y |
| Liu 2009 | Y | UC | N | Y | N | N |
| Pollak 2017 | Y | Y | N | Y | Y | Y |
| **Diuretics** |  |  |  |  |  |  |
| Murray 1997 | UC | N | N | Y | Y | Y |
| Almeida 2011 | Y | Y | UC | Y | N | Y |
| **Statins** |  |  |  |  |  |  |
| Wiwanitkit 2002 | UC | Y | Y | Y | Y | Y |
| Liu 2010 | Y | N | N | Y | Y | N |
| Kim 2010 | Y | Y | Y | Y | Y | Y |
| Kim 2013 | Y | UC | N | Y | Y | Y |
| Lee 2017 | Y | UC | N | Y | Y | N |
| Kim 2020 | UC | UC | N | Y | Y | N |

Y: low risk of bias; N: high risk of bias; UC: uncertainty about bias or lack of necessary information.

Supplementary Table 1. Risk assessment of bias in included studies

Supplementary Table 1 (b) Risk assessment of bias in non-randomized clinical studies

| Author / Year  （Classification by drug） | Confounding bias | selection bias | Bias in measurement classification of interventions | Bias due to deviations from intended interventions | Bias due to missing data | Bias in measurement of outcomes | Bias in selection of the reported result |
| --- | --- | --- | --- | --- | --- | --- | --- |
| **ACE inhibitors or ARBs** |  |  |  |  |  |  |  |
| Leclerc 2017 | M | M | L | L | M | S | L |
| Huang（I）2022 | L | M | L | L | M | M | L |
| **Anticoagulants** |  |  |  |  |  |  |  |
| Kwong 2012 | M | L | M | M | L | M | L |
| Hellfritzsch 2016 | L | L | M | L | L | L | L |
| Leclerc 2018 | L | L | M | L | M | M | L |
| Grampp 2015 | L | L | L | L | M | M | M |
| Desai 2020 | M | L | L | L | M | M | L |
| Fantoni 2021 | L | M | L | L | M | L | L |
| Feng 2009 | L | M | L | L | L | L | L |
| **Antiplatelet agents** |  |  |  |  |  |  |  |
| Park 2012 | M | L | L | M | M | M | L |
| Kovacic 2014 | L | L | L | L | L | M | M |
| Westphal 2022 | L | L | L | L | L | L | L |
| Ko 2018 | M | S | L | L | M | M | L |
| Leclerc 2019 | M | M | S | L | S | L | M |
| Patsourakos2020 | L | M | L | L | L | L | L |
| **Beta-blockers** |  |  |  |  |  |  |  |
| Ahrens 2007 | M | L | S | S | M | M | M |
| Chanchai 2018 | M | M | L | L | L | L | L |
| Huang(M) 2022 | M | M | L | L | M | L | L |
| Aretha 2020 | L | L | L | M | L | L | L |
| **Calcium channel blockers** |  |  |  |  |  |  |  |
| Desai2019 | M | M | L | L | M | L | L |
| Huang（A）2022 | L | L | L | L | M | M | M |
| Tung 2020 | NI | M | L | L | NI | NI | L |
| Lee 2022 | M | S | M | M | L | NI | L |
| Tung2022 | M | NI | L | L | M | NI | L |
| **Diuretics** |  |  |  |  |  |  |  |
| Martin 1984 | L | L | L | L | NI | L | L |
| Pan 1984 | L | L | L | L | NI | L | L |
| **Statins** |  |  |  |  |  |  |  |
| Corrao 2014 | M | L | NI | M | M | L | L |
| Gagne 2014 | L | L | M | M | M | M | L |
| Jackevicius 2016 | L | L | L | NI | NI | L | L |
| Sicras-Mainar2018 | NI | M | M | NI | M | L | L |
| Manasirisuk 2021 | L | L | NI | NI | L | M | L |

L: Low risk; M: Moderate risk; S: Serious risk; C: Critical risk; NI: No information.
